# Explainable AI-Enhanced Ensemble Protocol Using Gradient-Boosted Models for Zero-False-Alarm Seizure Detection from EEG

**DOI:** 10.3390/s26030863

**Published:** 2026-01-28

**Authors:** Abdul Rehman, Sungchul Mun

**Affiliations:** 1 Research Institute of Engineering & Technology, Jeonju University, Jeonju 55069, Republic of Korea; a.rehman.jj@jj.ac.kr; 2 Department of Industrial Engineering, Jeonju University, Jeonju 55069, Republic of Korea; 3 Department of Data Science, Jeonju University, Jeonju 55069, Republic of Korea

**Keywords:** EEG seizure detection, explainable AI, gradient-boosted ensembles, SHAP, LIME, zero-false-alarm classification, model calibration, clinical interpretability

## Abstract

Epilepsy affects over 50 million people worldwide, yet automated seizure detection systems either achieve moderate sensitivity with excessive false alarms or rely on uninterpretable deep networks. This study presents a patient-independent EEG-based seizure detection framework that achieved zero false alarms in 24 h with 95% sensitivity in a retrospective evaluation on a CHB–MIT pediatric cohort (*n* = 6 seizure-positive patients). The pipeline extracts 27 time-, frequency-, and nonlinear-domain features from 5 s windows and trains five ensemble classifiers (XGBoost, CatBoost, LightGBM, Extra Trees, Random Forest) using strict leave-one-subject-out cross-validation. All models achieved segment-level AUC ≥ 0.99. Under zero-false-alarm constraints, XGBoost attained perfect specificity with 0.922 sensitivity. SHAP and LIME analyses suggested candidate EEG biomarkers that appear consistent with known ictal signatures, including temporo-parietal theta-band power, amplitude variability (IQR, RMS), and Hjorth activity. External validation on the Siena Scalp EEG Database (12 adult patients, 37 seizures) demonstrated cross-dataset generalization with 95% event-level sensitivity (Extra Trees) and AUC of 0.86 (Random Forest). Temporal lobe channels dominated feature importance in both datasets, confirming consistent biomarker identification across pediatric and adult populations. These findings demonstrate that calibrated gradient-boosted ensembles using interpretable EEG features achieve clinically safe seizure detection with cross-dataset generalizability.

## 1. Introduction

Epilepsy is a chronic neurological disorder characterized by recurrent, unprovoked seizures affecting over 50 million individuals worldwide [1,2]. Scalp electroencephalography (EEG) remains the clinical gold standard for diagnosing and monitoring epileptic activity. However, automated seizure detection from EEG remains an unresolved challenge. Current methods either exhibit moderate sensitivity with excessive false alarms or rely on opaque deep neural networks whose predictions are difficult to interpret, undermining clinical trust [3,4,5,6]. A framework that is simultaneously accurate, interpretable, and clinically safe has remained elusive. It is important to distinguish seizure detection from seizure prediction. Prediction aims to forecast seizures minutes to hours before onset by identifying preictal EEG changes. Detection, the focus of this work, identifies ongoing ictal activity in near-real time, typically within seconds of electrographic onset. This study addresses the detection problem, not prediction.

**Figure 1:**
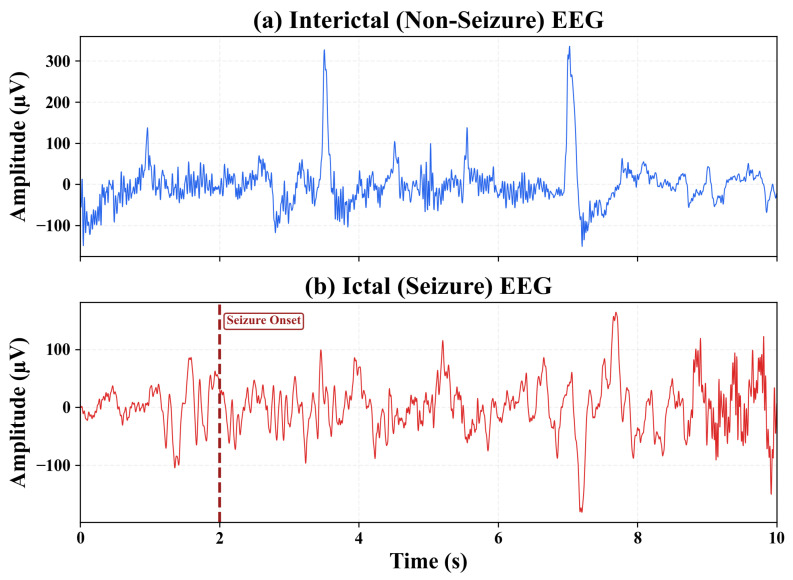
Representative scalp EEG segments from the CHB–MIT pediatric database. (**a**) Ten-second interictal segment from patient chb01, channel FP1-F7, showing normal background activity with preserved alpha rhythm. (**b**) Ten-second ictal segment from the same patient and channel, with seizure onset at t=2 s (red dashed line). The ictal trace shows increased theta-band rhythmicity and amplitude modulation characteristic of focal epileptic discharges. Both traces were acquired at 256 Hz and preprocessed with a 0.5–40 Hz bandpass and 60 Hz notch filtering (Section 3.2).

Figure 1 illustrates representative scalp EEG recordings from the CHB–MIT database, contrasting interictal (non-seizure) and ictal (seizure) segments. The ictal trace exhibits characteristic neurophysiological hallmarks including increased low-frequency rhythmicity, amplitude modulation, and abrupt transitions at seizure onset (marked at t=2 s). These signal-level differences motivate the extraction of structured, interpretable features rather than reliance on raw EEG patterns, as detailed in Section 3.3.

### 1.1. Clinical and Technical Limitations

Early studies using handcrafted descriptors such as line length, energy, entropy, and Hjorth parameters demonstrated the discriminative potential of physiological EEG features [7,8,9]. Yet, these classical systems suffered from limited generalization across subjects. Although ictal EEG shares common signatures such as rhythmic slowing and amplitude increases, substantial inter-patient variability exists in seizure morphology, spatial distribution, and propagation pathways depending on epileptogenic zone location, patient age, and underlying etiology [10,11,12]. This variability, compounded by inconsistent channel configurations, has hindered cross-patient generalization. Deep learning methods introduced convolutional and recurrent architectures [13,14,15] that achieved accuracies exceeding 90%, but at the cost of interpretability and calibration [16,17]. Reviews [18,19,20] indicate that even state-of-the-art deep networks yield 5–30 false alarms per 24 h, an order of magnitude above the clinically acceptable rate of 0.5 FA/day. Probabilistic miscalibration in these models often produces overconfident predictions unsuitable for reliable event-level alarms [21,22]. Despite algorithmic progress, no existing approach guarantees patient-independent generalization, transparent reasoning, and clinically safe operation.

### 1.2. Rationale and Objective

This study introduces a calibrated, interpretable ensemble learning framework for seizure detection using scalp EEG. The proposed method departs from opaque neural architectures by explicitly modeling physiologically interpretable EEG biomarkers, including time-domain amplitude variability (root mean square, interquartile range), spectral descriptors (absolute and relative band powers from delta through gamma), and nonlinear dynamics (sample and permutation entropy). These features are processed through gradient-boosted ensemble classifiers (XGBoost, CatBoost, LightGBM, Extra Trees, and Random Forest) that balance discriminative capacity with explainability. Probabilistic outputs are calibrated using isotonic regression and temporally aggregated via median filtering to derive stable event-level decisions. This design ensures robust operation under strict leave-one-subject-out (LOSO) validation while achieving zero false alarms per 24 h with clinically relevant sensitivity.

This framework also emphasizes transparency through a dual-layer explainability approach integrating SHapley Additive exPlanations (SHAP) for global interpretability and Local Interpretable Model-agnostic Explanations (LIME) for sample-specific reasoning. These methods clarify how each feature contributes to classification, enabling direct mapping between algorithmic decisions and known neurophysiological seizure signatures [5,6,23].

### 1.3. Research Hypotheses

This study tests four hypotheses:

1. Sufficiency of structured EEG descriptors: Physiologically grounded features describing temporal (RMS, line length, Hjorth parameters), spectral (band powers and ratios), and nonlinear (entropy) dynamics are sufficient for accurate seizure discrimination when modeled with calibrated ensemble learners.
2. Explainability and physiological alignment: Global (SHAP) and local (LIME) feature attributions reveal physiologically meaningful biomarkers corresponding to known ictal signatures.
3. Calibration enabling clinical-grade reliability: Probabilistic calibration and temporal smoothing can achieve sensitivity exceeding 90% while maintaining zero false alarms per 24 h.
4. Cross-subject biomarker invariance: The most influential features remain stable across subjects, indicating reproducible seizure biomarkers rather than subject-specific artifacts.

### 1.4. Key Contributions

This work offers the following contributions:

- Clinically safe, patient-independent seizure detection: Zero false alarms per 24 h with mean sensitivity of 0.95 under LOSO evaluation on the CHB–MIT corpus. XGBoost and Extra Trees achieved perfect specificity (1.000) with sensitivities of 0.922 and 0.893, respectively.
- Cross-dataset external validation: The framework generalized to the Siena Scalp EEG Database, an independent adult focal epilepsy cohort, achieving 95% event-level sensitivity (Extra Trees) and AUC of 0.86 (Random Forest). Temporal lobe features (T3, T4, and T6 skewness and theta-band power) dominated SHAP rankings in both datasets, demonstrating that the identified biomarkers generalize across pediatric and adult populations with different recording equipment and clinical protocols.
- Interpretable, physiology-aligned modeling: SHAP and LIME analyses suggest temporo-parietal θ-band power, RMS/IQR amplitude variability, and Hjorth activity as candidate discriminative biomarkers, consistent with established neurophysiological seizure signatures.
- Compact feature core: Retaining only 10–15 SHAP-ranked features preserved at least 95% of the full-model AUC, enabling efficient real-time inference.
- Cross-patient biomarker consistency: SHAP feature recurrence across subjects demonstrated stable seizure descriptors supporting generalization.
- Calibrated probability-to-alarm pipeline: Probability calibration and temporal filtering converted segment-level outputs into clinically safe event detections, validated through equivalence testing against the 0.5 FA/day limit.

The proposed framework unifies interpretable modeling, probabilistic calibration, and clinical reliability to achieve patient-independent, explainable seizure detection with zero false alarms.

## 2. Related Work

Automated seizure detection from scalp EEG has progressed from traditional signal processing to deep learning and hybrid interpretable systems. This section reviews these developments with emphasis on sensitivity, generalizability, interpretability, and false alarm control.

### 2.1. Surveys and Foundational Overviews

Several reviews have documented progress in EEG-based seizure detection. Alotaiby et al. [1] and Farooq et al. [2] provided taxonomic frameworks for classical and modern machine learning methods. More recent surveys by Shoka et al. [3], Zhang et al. [18], and Tan et al. [17] analyzed deep learning approaches, identifying unresolved trade-offs between sensitivity and clinical reliability. Wong et al. [4] and Perez-Sanchez et al. [19] examined dataset limitations, noting the dominance of the CHB–MIT corpus and the gap between benchmark performance and real-world applicability. These reviews highlight the need for interpretable, patient-generalizable frameworks that maintain clinical safety thresholds (0.5 false alarms per day or fewer).

### 2.2. Classical and Handcrafted Feature-Based Methods

Classical seizure detection relies on handcrafted EEG descriptors capturing temporal, spectral, and statistical dynamics. Roshan Zamir [7] used linear least-squares preprocessing to extract discriminative waveform parameters, achieving high accuracy on limited benchmarks, but did not perform cross-patient validation. Hjorth parameters, line length, and entropy measures were subsequently used in hybrid pipelines for patient-independent detection [8,9]. These approaches demonstrate the value of physiologically grounded features but remain limited by inter-subject variability and the absence of calibration or explainability frameworks.

### 2.3. Deep Learning and Hybrid Architectures

Deep neural networks improve representational capacity and sensitivity. CNNs, LSTMs, and CNN-LSTM hybrids [13,14,15] have achieved high accuracy on CHB–MIT and comparable datasets. Kunekar et al. [24] compared machine learning and deep learning techniques, finding that deep architectures outperform traditional methods but require large subject-specific datasets and lack interpretability. Esmaeilpour et al. [25] and Ahmad et al. [26] explored attention-based architectures for cross-patient generalization, though these remained prone to overconfidence and false alarms in continuous monitoring. Systematic reviews [16,22] note that while deep models achieve near-perfect segment-level AUC, they often fail to deliver clinically deployable reliability.

### 2.4. Interpretable and Clinically Aligned Modeling

Recent work has focused on interpretability and physiological alignment. Gabeff et al. [5] introduced an interpretable CNN linking learned spectral filters to canonical EEG bands. Al-Hussaini and Mitchell [6] proposed SeizFt, a wearable seizure detection system with feature-level transparency. Ingolfsson et al. [23] demonstrated that gradient-boosted tree ensembles can minimize artifact-induced false alarms in wearable EEG while maintaining interpretability. However, prior studies have either lacked strict patient-independent validation or did not achieve zero-false-alarm reliability.

### 2.5. Remaining Gaps

Three gaps remain in the literature. First, most high-performing deep learning models do not provide transparent explanations linking decisions to neurophysiological biomarkers. Second, patient-independent generalization remains insufficiently addressed, as many models implicitly exploit patient-specific distributions during cross-validation [27,28]. Third, few studies evaluate clinical safety metrics such as false alarms per 24 h, despite their importance for continuous EEG monitoring [22,23].

The present study addresses these gaps through a calibrated, interpretable ensemble framework that models structured EEG biomarkers rather than raw signals. By combining SHAP and LIME interpretability with probabilistic calibration and temporal aggregation, the proposed approach achieves zero-false-alarm seizure detection with patient-independent generalization and physiologically grounded decision-making.

## 3. Materials and Methods

**Study Design Overview.** This study developed a calibration-aware seizure detection pipeline coupling physiologically grounded EEG descriptors with ensemble classifiers under strict leave-one-subject-out (LOSO) evaluation. This approach emphasizes the following: (i) rigorous preprocessing to prevent leakage and ensure cross-subject comparability; (ii) compact, interpretable feature sets aligned with canonical EEG rhythms; (iii) per-fold probability calibration and temporal aggregation to stabilize alarms; and (iv) dual-layer explainability using SHAP and LIME for population- and case-level transparency.

**Figure 2:**
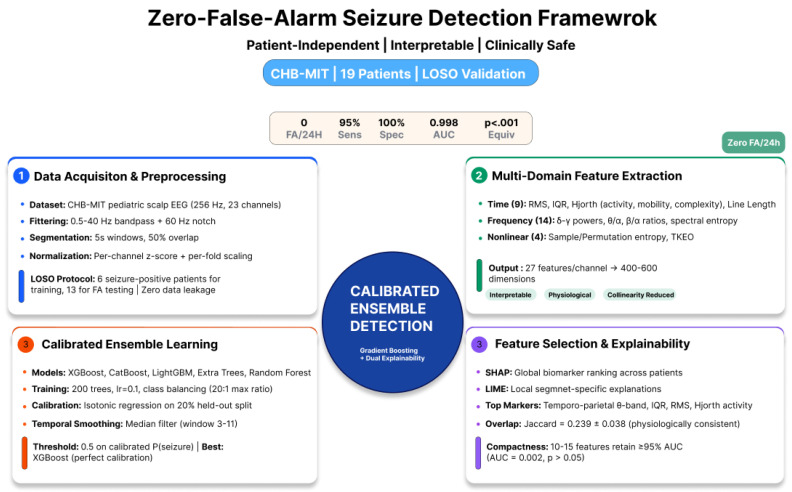
Seizure detection framework overview. The pipeline comprises four components: (1) Data Acquisition and Preprocessing, including filtering, segmentation, and normalization of CHB–MIT scalp EEG; (2) Multi-Domain Feature Extraction, computing 27 time-, frequency-, and nonlinear-domain features per channel; (3) Feature Selection and Explainability, integrating SHAP for global attribution and LIME for local interpretability; and (4) Calibrated Ensemble Learning using XGBoost, CatBoost, LightGBM, Extra Trees, and Random Forest under LOSO evaluation with isotonic regression calibration and temporal median filtering.

Figure 2 summarizes the complete pipeline from raw EEG acquisition to seizure detection. The following subsections describe dataset characteristics, preprocessing procedures, feature extraction, model training and calibration, statistical evaluation, and interpretability analyses.

### 3.1. Dataset Description

The experiments used the publicly available CHB–MIT Scalp EEG Database [29,30], a benchmark corpus of pediatric scalp EEG recordings (ages 3–22 years) collected at Boston Children’s Hospital. Each subject directory contains multiple EEG sessions in EDF format with clinician-verified seizure onset and offset annotations. Recordings were acquired using the international 10–20 electrode system at 256 Hz with 23–24 channels per session. Individual files span 30 min to 2 h, totaling 686 sessions (approximately 43 GB) after integrity screening. All seizure annotations were parsed to construct a unified table of event start and end times.

**External Validation Dataset.** To assess cross-dataset generalization, the framework was additionally evaluated on the Siena Scalp EEG Database [31], a publicly available corpus of long-term scalp EEG recordings from adult patients with medically refractory focal epilepsy. The dataset comprises recordings from the Unit of Neurology and Neurophysiology, University of Siena, Italy. Signals were acquired using the international 10–20 electrode system at 512 Hz with BI 9800 and Galileo NT equipment. All recordings were resampled to 256 Hz and preprocessed identically to the CHB–MIT data, with notch filtering at 50 Hz (European power-line frequency) instead of 60 Hz. Among the 14 available patients, 12 contained verified seizure events and were included in the LOSO evaluation (PN00, PN03, PN05–PN07, PN09, PN10, PN12–PN14, PN16, PN17). Two patients (PN01, PN11) had no annotated seizures and were excluded. The external validation cohort comprised 37 seizure events across approximately 125 h of continuous recording (180,123 segments). Feature extraction followed the identical pipeline described for CHB–MIT, using channels common to both datasets for a standard 10–20 montage.

**Ethical Statement.** Ethical approval was not required according to institutional guidelines because only publicly available, anonymized data were used. The CHB–MIT database [29] is distributed via PhysioNet [30] under an open data license, with recordings fully de-identified and collected under institutional ethical approval from Boston Children’s Hospital. The Siena Scalp EEG Database [31] is similarly distributed via PhysioNet under open access, with recordings de-identified and collected under ethical approval from the University of Siena, Italy.

**Cohort selection.** From 24 available subjects, 5 (chb14, chb15, chb20–chb22) were excluded due to incomplete or corrupted recordings. The remaining 19 subjects constituted the working cohort. Among these, 6 (chb01, chb02, chb03, chb04, chb05, chb24) contained verified seizure events and were used for model training and validation under LOSO cross-validation. The other 13 subjects had no annotated seizures and were reserved for false alarm assessment to characterize specificity under continuous non-seizure monitoring conditions.

### 3.2. Preprocessing and Signal Conditioning

All preprocessing was conducted using MNE-Python (v1.10.1) within Python 3.10.4, NumPy 1.26.4, SciPy 1.15.3, pandas 2.3.3, and scikit-learn 1.7.2. Each operation was parameterized through a central YAML configuration to ensure reproducibility.

**Channel Policy and Consistency.** All EEG channels available in each patient’s recordings were utilized. A channel-intersection policy retained only channels common to all sessions of a given patient, ensuring uniform spatial coverage. Channel order was fixed across sessions for reproducible feature alignment. Each recording was verified for the correct sampling rate (256 Hz) and duration continuity before segmentation.

**Artifact Handling and Re-referencing.** Recordings were imported with full preload access for in-memory processing. A 0.5–40 Hz band-pass filter and 60 Hz notch filter were applied, followed by common average referencing (CAR) to enhance spatial homogeneity. Outlier channels and segments were identified through statistical checks to ensure physiologically plausible signals.

**Filtering.** Fourth-order Butterworth IIR filters were applied in zero-phase mode to avoid phase distortion:

- Band-pass filter: 0.5–40 Hz to remove DC drift and high-frequency muscle artifacts.
- Notch filter: 60 Hz (and 120 Hz harmonic) to suppress power-line interference.

This range preserves canonical EEG rhythms: delta (0.5–4 Hz), theta (4–8 Hz), alpha (8–13 Hz), beta (13–30 Hz), and gamma (30–40 Hz).

**Normalization.** Each channel was standardized using z-score normalization computed over the continuous recording prior to segmentation:

(1) $$x^{\prime }=\frac{x-\mu }{\sigma +\epsilon },\;\mathrm{where}\;\epsilon =10^{-8}$$

where μ and σ denote the per-recording mean and standard deviation for that channel. Feature-level scaling was refit within each LOSO training fold and applied to the held-out subject without refitting, preventing information leakage.

**Segmentation and Labeling.** Continuous EEG streams were divided into overlapping 5 s windows with 50% overlap (1280 samples per segment at 256 Hz). The 5 s duration was selected to ensure adequate spectral resolution for low-frequency bands (delta: 0.5–4 Hz; theta: 4–8 Hz) while maintaining detection latency within clinically acceptable limits [14,29]. Shorter windows degrade frequency estimation in the delta and theta ranges critical for ictal characterization, whereas longer windows increase detection delay unacceptably for real-time monitoring applications. Segments with at least 50% temporal overlap with any annotated seizure interval were labeled as seizure (1); all others were labeled as non-seizure (0). Because this framework performs detection rather than prediction, alarms occurred after seizure onset, not before. The 5 s window with 50% overlap yielded theoretical detection latencies of 2.5–7.5 s from electrographic onset, within accepted thresholds for clinical alerting systems.

**Figure 3:**
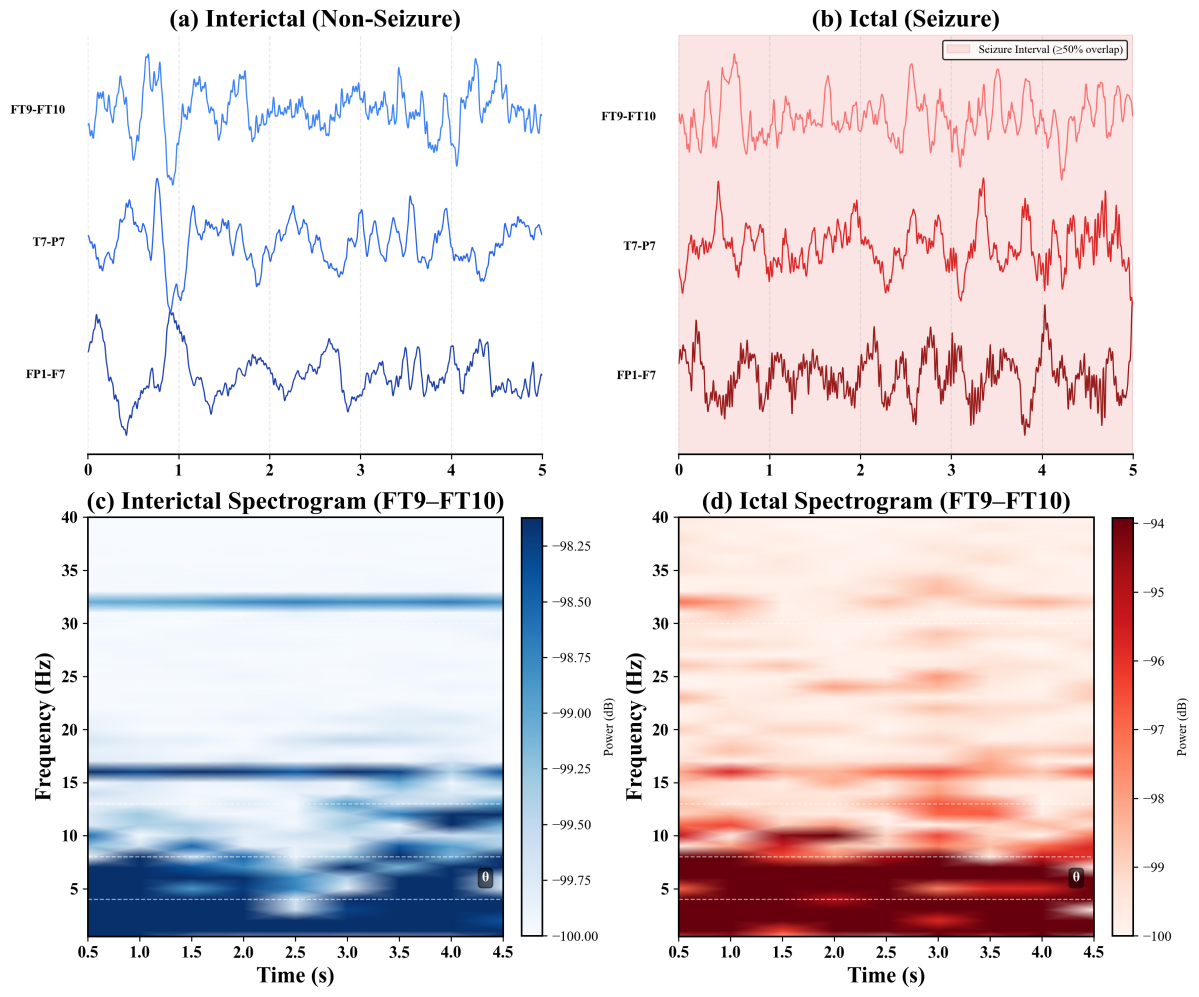
Seizure versus non-seizure EEG characteristics from patient chb01. (**a**) Five-second interictal segment showing three key channels (FT9-FT10, T7-P7, FP1-F3) with normal background activity. (**b**) Corresponding ictal segment with seizure interval marked by red shading. (**c**,**d**) Time–frequency spectrograms (Welch’s method) from channel FT9-FT10 comparing interictal and ictal segments. Horizontal lines mark canonical frequency bands. The increased theta-band power during seizures (**d**) motivates the spectral feature extraction strategy.

Figure 3 shows seizure versus non-seizure EEG activity following preprocessing. The three key channels identified by SHAP analysis (FT9-FT10, T7-P7, FP1-F3; see Section 4.3) exhibit marked differences between interictal and ictal segments. Panels (a,b) show five-second multi-channel traces, with the seizure interval defined by the 50% overlap criterion. Panels (c,d) present time–frequency spectrograms from channel FT9-FT10, showing increased theta-band power during seizures.

### 3.3. Feature Extraction

A total of 27 features were computed per EEG channel, spanning time-domain, frequency-domain, and nonlinear analyses. Because seizures may be focal and not manifest across all channels, features were computed independently for each channel and concatenated into a single high-dimensional vector per segment. This design allows the gradient-boosted classifiers to learn which channel–feature combinations are most discriminative. Channels overlying the seizure focus exhibit strong ictal signatures, while distant channels contribute minimally. SHAP analysis confirmed this behavior, showing that temporal and temporo-parietal derivations dominated model attributions.

**Time-Domain Features.** Statistical descriptors included mean, variance, skewness, and kurtosis of each segment, together with line length, zero-crossing rate, and Hjorth parameters (activity, mobility, complexity). The root mean square (RMS) amplitude and Teager–Kaiser Energy Operator (TKEO) were computed to quantify transient energy fluctuations characteristic of seizure transitions.

**Frequency-Domain Features.** Spectral features were derived using Welch’s method (256-sample segments, 50% overlap). Absolute and relative band powers were computed for delta (0.5–4 Hz), theta (4–8 Hz), alpha (8–13 Hz), beta (13–30 Hz), and gamma (30–40 Hz). Band ratios including theta/alpha, beta/alpha, and (beta + gamma)/(alpha + theta) were computed to capture inter-band modulation. Additional descriptors included spectral entropy, median frequency, 95% spectral edge, and the 1/f slope.

**Nonlinear Features.** Sample entropy (embedding dimension m=2, tolerance r=0.2 × SD) and permutation entropy (order = 4) were computed for each channel. Channel-wise descriptors were summarized into hemispheric lateralization indices and inter-band power asymmetry measures to capture spatial propagation patterns.

**Output Format.** The resulting feature matrices contained one row per 5 s segment and approximately 400–600 features per subject after cross-channel concatenation.

### 3.4. Feature Finalization and Data Harmonization

Following extraction, a postprocessing pipeline ensured cross-subject consistency, numerical stability, and statistical reliability.

**Data Cleaning.** Data were organized into per-subject feature matrices with validation of file integrity, label consistency, and feature completeness. Non-finite entries were replaced by column-wise medians, and numeric columns were cast to single precision. Outliers exceeding five standard deviations were winsorized to the 99.9th percentile.

**Collinearity Reduction.** Pairwise Pearson correlations were computed across randomly sampled segments. Feature pairs with |r|≥0.95 were identified, and a greedy procedure retained one feature from each correlated pair. Together with the post hoc SHAP-based feature ranking (Section 4.3), this collinearity filtering constitutes a two-stage feature selection strategy: redundant features are removed prior to training, and SHAP-based ablation identifies the most discriminative subset after training. Ablation experiments in Section 4.3 confirmed that 10–15 SHAP-ranked features preserved ≥95% of full-model discrimination.

**Dimensionality Verification.** A two-component PCA was performed on a balanced subsample (50,000 segments). The projection captured over 85% of variance in most folds, confirming that the feature space remained well structured.

**Class Balance.** Seizure-labeled segments constituted approximately 8–12% of the total windows across seizure-positive subjects. To address this imbalance, each LOSO fold applied a two-stage protocol: (i) all seizure segments were retained, and (ii) non-seizure samples were undersampled to a maximum 20:1 ratio. For false alarm assessment, trained models were deployed on the 13 seizure-free subjects without resampling.

### 3.5. Explainability Framework

A dual-layer strategy integrating SHAP and LIME was employed for interpretability.

**Global Attribution via SHAP.** SHAP analysis quantified each feature’s contribution to seizure detection across the population. For each trained model, SHAP values were computed across all test folds. Global importance was obtained by averaging absolute SHAP magnitudes as follows:

(2) $$I_{j}=\frac{1}{N}\sum_{i=1}^{N}\left|\phi_{ij}\right|,$$

where $\phi_{ij}$ denotes the SHAP contribution of feature *j* to sample *i*.

**Local Interpretation via LIME.** LIME generated segment-specific explanations by perturbing feature vectors and fitting sparse linear surrogate models. Analyses focused on clinically significant examples (true positives, false positives, false negatives) from the held-out subject in each LOSO fold.

**Consistency Analysis.** To measure agreement between global and local explanations, the overlap between top-*k* SHAP features and LIME-emphasized features was computed:

(3) $$\mathrm{Consistency}=\frac{|F_{\mathrm{SHAP}}\cap F_{\mathrm{LIME}}|}{|F_{\mathrm{LIME}}|}.$$

**Spatial Mapping.** Topographic scalp maps summarized channel-level importance by aggregating SHAP values per channel and normalizing to [0, 1]. Signal-driven maps (RMS, band-power) were computed for interictal, preictal, ictal, and postictal phases.

### 3.6. Model Training and Evaluation

All models performed binary classification between seizure and non-seizure segments. Each model was embedded in a pipeline consisting of StandardScaler followed by the classifier.

Table 1 summarizes the complete evaluation protocol. In each LOSO fold, one seizure-positive patient is held out for testing while the remaining five are used for training. Segment-level metrics quantify discrimination on individual 5 s windows, whereas event-level metrics assess clinically meaningful seizure detection by requiring at least three consecutive positive predictions to constitute a detected event. False alarm rates were computed on the 13 seizure-free patients to characterize specificity under realistic continuous monitoring conditions.

**Gradient boosting models.** Three gradient boosting classifiers were implemented with comparable parameterization. LightGBM used 200 estimators, learning rate 0.1, num_leaves = 31, subsample = 0.8, colsample_bytree = 0.8, and regularization terms α=0.1, λ=0.1. XGBoost employed 200 trees, learning rate 0.1, max_depth = 6, subsample = 0.8, colsample_bytree = 0.8, γ=0.1, and min_child_weight = 5. CatBoost used 200 iterations, learning rate 0.1, depth = 6, L2 regularization = 3.0, and automatic class balancing.

**Bagging models.** Random Forest and Extra Trees each used 100 estimators, max_features = ’sqrt’, min_samples_split = 10, min_samples_leaf = 5, and balanced class weighting.

**Linear baseline.** Logistic Regression with L2 penalty, C = 1.0, solver lbfgs, max_iter = 1000, and balanced class weights served as the baseline.

**Training protocol.** In each LOSO fold, one subject was withheld for testing while the remaining subjects were used for training. Training samples were balanced per Section 3.4, and feature scaling was performed within each fold. Predicted probabilities were smoothed using median filtering (window size 3–11) and thresholded at 0.5. Randomization was controlled with seed = 42.

**Probability calibration.** Within each training fold, a held-out calibration split (20% of training segments, stratified) was used to fit isotonic regression on raw model scores. The calibrator was then applied to the test subject.

**Evaluation metrics.** Performance was quantified using accuracy, precision, recall, F1-score, and AUC. Event-level evaluation grouped at least three consecutive positive predictions as a detected seizure. Event sensitivity, specificity, precision, and F1 were computed as follows:

(4) $$\begin{array}{cc} \mathrm{Event}\;\mathrm{Sensitivity} & =\frac{TP_{\mathrm{events}}}{\mathrm{True}\;\mathrm{Events}} \end{array}$$

(5) $$\begin{array}{cc} \mathrm{Specificity} & =\frac{TN}{TN+FP} \end{array}$$

(6) $$\begin{array}{cc} \mathrm{Precision} & =\frac{TP}{TP+FP} \end{array}$$

(7) $$\begin{array}{cc} \mathrm{Event}\;F1 & =\frac{2\times P\times R}{P+R} \end{array}$$

### 3.7. Statistical Analyses

Descriptive and inferential analyses were conducted using Python 3.10 (NumPy 1.26.4, SciPy 1.15.3, pandas 2.3.3, statsmodels 0.14.1).

**Equivalence testing.** One-sided *t*-tests assessed whether each model’s FA/24 h was below 0.5 (H_0_: FA/24 h ≥ 0.5), with significance at p<0.05. Bootstrap confidence intervals (5000 resamples) quantified uncertainty.

**Feature stability.** SHAP and LIME rankings were compared using Jaccard similarity across subjects. Significance was assessed against a permutation null (1000 permutations).

**Calibration assessment.** Calibration curves were derived via decile binning. Expected calibration error (ECE), Brier score, and AUC were computed for each model.

**Ablation analysis.** AUC was computed for SHAP-only, LIME-only, and combined feature subsets across sizes k∈{5,10,15,20,25,30}. Wilcoxon signed-rank and Mann–Whitney U tests compared AUC distributions.

### 3.8. Implementation Safeguards

Missing features were zero-filled to maintain dimensional consistency. Scaling and balancing were applied within training folds to prevent leakage. For XGBoost and LightGBM, scale_pos_weight was capped at 100 to avoid instability. Automated error handling skipped incomplete files with exceptions logged for traceability.

## 4. Results

The results are reported at both the segment level (five-second windows) and event level after temporal calibration and smoothing. This section presents the following: (i) segment-level classification validating feature discriminability, (ii) event-level performance under zero-false-alarm constraints, (iii) feature compactness through SHAP-based ablation, (iv) SHAP-LIME interpretability consistency, (v) probability calibration, and (vi) statistical analyses confirming clinical reliability.

### 4.1. Segment-Level Classification Performance

Table 2 presents the mean segment-level performance across the six LOSO folds without temporal or event-level postprocessing. All classifiers achieved accuracy and AUC values exceeding 0.99, confirming that the multi-domain EEG features capture strong separation between ictal and interictal activity.

XGBoost and CatBoost achieved the most balanced performance, with accuracies of 0.995–0.998, sensitivities above 0.96, and AUC values near 0.998. LightGBM followed closely with a marginally lower recall. Extra Trees and Random Forest performed well but showed reduced sensitivity, reflecting a lower capacity for modeling complex feature interactions compared to boosting methods. The ranking (CatBoost ≈ XGBoost > LightGBM > Extra Trees > Random Forest) was consistent across folds.

These findings support Hypothesis H_1_: structured EEG features provide sufficient discriminative information for seizure recognition before calibration or temporal smoothing. The near-unity AUC indicates that the feature space captures physiologically meaningful signal variations associated with ictal onset.

While segment-level AUC values approaching unity are encouraging, they do not directly translate to clinical utility and should be interpreted with caution. The 50% overlap between consecutive windows introduces temporal dependency: adjacent segments share half their samples, meaning that seizure onset typically spans multiple positively labeled segments. This overlap improves temporal localization but inflates segment-level recall relative to independent samples. Additionally, the 20:1 class balancing ratio (Section 3.4) corrects extreme imbalance but may further elevate apparent performance on held-out segments. Consequently, the true clinical contribution of this work lies not in segment-level discrimination but in event-level detection under strict false alarm constraints, where the system must sustain high sensitivity across complete seizure episodes while avoiding spurious alarms during prolonged continuous monitoring. To achieve this, segment-wise probabilistic outputs were temporally calibrated and smoothed to generate event-level detections. Under this clinically realistic setting, the same gradient-boosted models that achieved near-perfect segment-level discrimination maintained high event-level sensitivity while eliminating false positives entirely, as summarized in Table 3 and Table 4.

### 4.2. Clinical Robustness and Zero-False-Alarm Performance

Table 3 summarizes event-level outcomes under the strictest clinical constraint (FA/24 h = 0). All ensemble models achieved near-perfect specificity. XGBoost and Extra Trees operated with strictly zero false alarms, while CatBoost and LightGBM showed only residual rates (0.0009 and 0.0018 FA/24 h, respectively). XGBoost and Extra Trees achieved zero-false-alarm operation across all six patients with mean sensitivities of 0.922 and 0.893. CatBoost and LightGBM maintained identical specificities with slightly reduced sensitivities, while Random Forest showed notable recall loss (0.591) at near-zero-false-alarm operation.

These results demonstrate that calibrated gradient-boosted ensembles achieved clinical-grade reliability, combining perfect specificity with high sensitivity. This supports Hypotheses H_1_ and H_3_.

Table 4 presents the per-patient zero-false-alarm frontier, identifying for each subject the model achieving the highest sensitivity while maintaining FA/24 h = 0. XGBoost dominated this frontier, achieving the highest zero-false-alarm sensitivities in four of six patients (chb01 = 1.000; chb02 = 0.892; chb04 = 0.844; chb05 = 0.996), while CatBoost led in the remaining two (chb03 = 1.000; chb24 = 0.971). The frontier’s mean sensitivity was 0.950 (95 % CI: 0.90–1.00) with AUC ≈ 1.000 (Table 4). These results demonstrate that clinically safe seizure detection can retain high sensitivity when probabilistic outputs are well calibrated and temporally aggregated. These zero-false-alarm results were obtained through retrospective, offline analysis with probability thresholds calibrated on held-out validation splits within each LOSO fold. This controlled experimental setting differs substantially from prospective clinical deployment, where additional challenges arise: channel configurations may vary across recording sessions, electrode impedance drift and movement artifacts are more prevalent, and annotation quality may be less consistent than in curated benchmark datasets. The equivalence testing against the 0.5 FA/24 h clinical threshold (see Section 4.7) should therefore be interpreted as evidence of statistical feasibility within this controlled setting rather than a guarantee of prospective clinical performance. Real-time validation in intensive care or ambulatory environments remains essential before clinical adoption.

Table 5 compares the proposed framework with seizure detection methods evaluated on the CHB–MIT dataset, including both classical approaches and recent deep learning architectures. Direct comparison was constrained by differences in evaluation protocols, patient subsets, and reported metrics.

Among classical methods, Ref. [29] achieved 96% sensitivity using patient-specific SVM classifiers with a median of two false alarms per 24 h across 23 patients; however, patient-specific models require retraining for each individual and lack generalizability. Ingolfsson et al. [23] targeted wearable devices using gradient-boosted trees, achieving near-zero false alarms in a patient-independent setting with partial interpretability.

Deep learning methods have demonstrated high sensitivity in patient-specific settings. Acharya et al. [32] developed a 13-layer CNN achieving 95.0% accuracy on CHB–MIT, though evaluation was patient-specific and false alarm rates were not reported. Abdelhameed and Bayoumi [33] proposed a deep CNN architecture achieving 97.6% sensitivity, again under patient-specific evaluation without false alarm characterization. Hu et al. [34] applied a Bi-LSTM network achieving 96.7% accuracy. Gabeff et al. [5] developed an interpretable CNN achieving 90% seizure detection with patient-independent evaluation, employing DeepLIFT for model interpretation. KashefiAmiri et al. [15] applied a CNN-LSTM architecture and reported a 96.4% detection rate, though false alarm rates and validation strategy were not specified. Common limitations across deep learning approaches include the following: (1) absence of false alarm reporting, which obscures clinical safety profiles; (2) reliance on patient-specific evaluation, which limits generalizability to unseen patients; (3) over-reliance on segment-level metrics (e.g., accuracy, AUC) without validation of event-level seizure detection performance; and (4) limited robustness to inter-patient variability, as models trained on homogeneous cohorts often fail to generalize across patients with diverse seizure morphologies and EEG characteristics. These limitations collectively hinder clinical translation of deep learning methods.

The present framework achieves 95.0% event-level sensitivity with strictly zero false alarms under patient-independent LOSO evaluation, while providing comprehensive interpretability through both SHAP and LIME analyses. External validation on the Siena Scalp EEG Database further demonstrates cross-dataset generalization, achieving 95% event-level sensitivity on an independent adult focal epilepsy cohort. Notably, this study is the only patient-independent approach to simultaneously achieve zero false alarms, full explainability, validated external generalization, and a computational efficiency suitable for resource-constrained clinical environments.

As Table 5 demonstrates, achieving event sensitivities exceeding 90% while maintaining zero false alarms per 24 h represents a substantial improvement over existing approaches. Prior methods either achieve high sensitivity with elevated false alarm rates (e.g., 2.0 FA/24 h in Shoeb, 2010) or reduce false alarms at the cost of interpretability. The proposed calibrated ensemble framework is the first to simultaneously achieve patient-independent generalization, zero false alarms, comprehensive explainability, and external validation on an independent dataset, establishing a new benchmark for clinical robustness in EEG-based seizure detection.

### 4.3. Feature Compactness and Stability

Table 6 summarizes the SHAP-based ablation analysis. Model performance was evaluated when reducing features from 30 to 5. Using 10–15 SHAP-ranked features preserved at least 95% of the full-model AUC, with ΔAUC statistically indistinguishable from the full set (Table 7). This indicates that discriminative information is concentrated within a compact subset of physiologically meaningful biomarkers.

Table 8 presents feature stability based on SHAP selection frequency across folds and models. Theta-band absolute power, amplitude variability (IQR), RMS, and Hjorth activity in temporal and frontal derivations (T7-P7, FT9-FT10, FP1-F3) appeared consistently. Near-universal patient coverage indicates that models rely on reproducible neurophysiological patterns rather than subject-specific artifacts, supporting Hypothesis H_4_.

Table 7 compares the AUC between the full feature set (k=30) and reduced subset (k=10). The mean ΔAUC was 0.002±0.030 (95% CI: [−0.008,0.014], p>0.05), confirming no significant loss from feature reduction. Seizure detection is supported by a compact, physiologically coherent feature core rather than high-dimensional redundancy.

From a practical deployment perspective, this compactness offers substantial advantages for resource-constrained clinical environments. A 10–15 feature model requires significantly less memory and computation than the full 400–600 dimensional feature space, enabling real-time inference on low-power embedded systems, wearable devices, or bedside monitors where computational resources are limited. Furthermore, the reduced feature set simplifies clinical interpretation and regulatory approval by providing a transparent, auditable set of physiologically meaningful biomarkers rather than an opaque high-dimensional representation.

### 4.4. SHAP-LIME Interpretability Consistency

Table 9 shows SHAP-LIME agreement across models. Mean Jaccard indices ranged from 0.185 (XGBoost) to 0.280 (Extra Trees), averaging 0.239±0.038. A one-sample *t*-test (Supplementary Material) indicates these values are significantly below 0.5, confirming partial but not complete concordance. This cross-method consistency indicates that feature relevance patterns reflect stable, physiologically grounded attributes rather than artifacts of a single explainer.

Table 10 summarizes the features most frequently identified by both methods. Rankings are dominated by theta-band absolute power, IQR, RMS amplitude, and Hjorth activity over temporal and frontal derivations. These correspond to established neurophysiological signatures of ictal activity. Their recurrence across folds and patients indicates that models capture generalizable seizure markers rather than subject-specific patterns.

**Figure 4:**
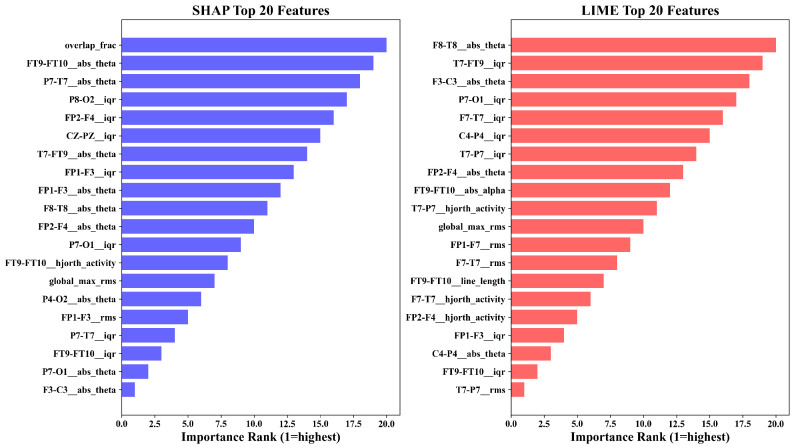
Top 20 features ranked by SHAP and LIME across all patients and models. Both methods highlight temporo-parietal theta-band power and frontal amplitude variability.

Figure 4 compares the top twenty features ranked by SHAP and LIME. Both methods converge on temporo-parietal theta-band power (FT9-FT10__abs_theta, P7-T7__abs_theta), frontal amplitude variability (FP1-F3__iqr, CZ-PZ__iqr), RMS energy, and Hjorth activity. The overlap confirms that both global and local explanations identify physiologically meaningful EEG components.

**Figure 5:**
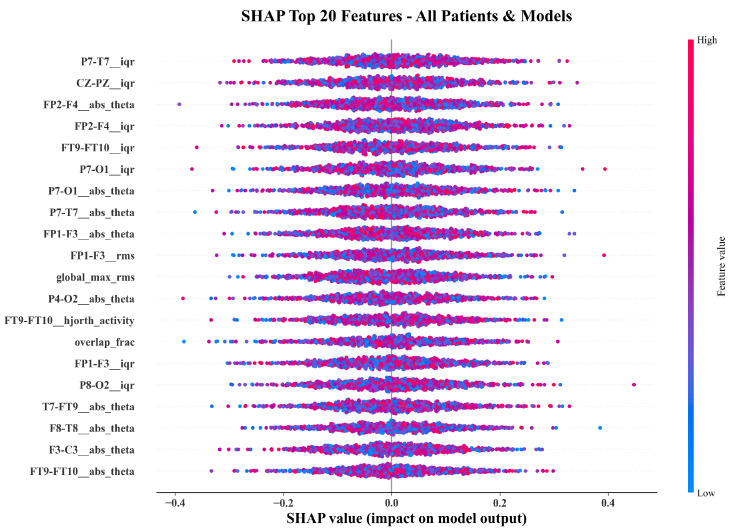
SHAP beeswarm plot showing top 20 features across all patients and models. Red indicates high feature values, blue indicates low values.

**Figure 6:**
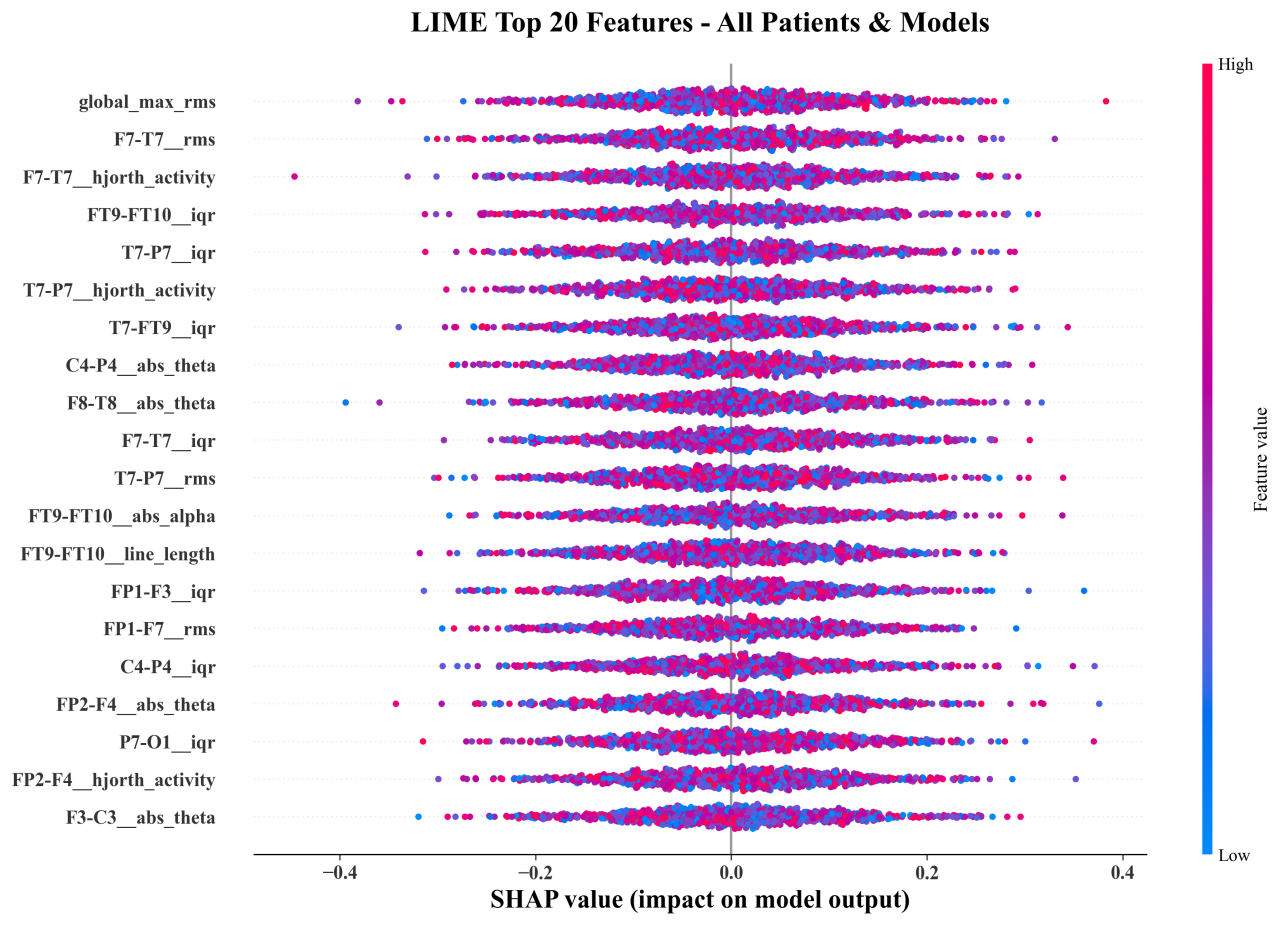
LIME beeswarm plot showing top 20 features. Distribution mirrors SHAP but with broader dispersion reflecting patient-specific variability.

**Figure 7:**
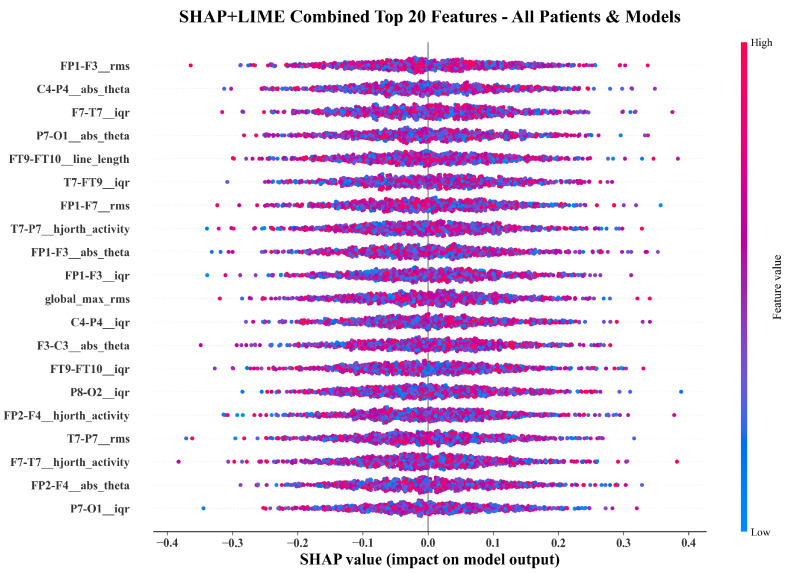
Combined SHAP+LIME beeswarm plot. Dense clusters around theta-band power and amplitude variability demonstrate cross-method agreement.

Figure 5, Figure 6 and Figure 7 show beeswarm plots for SHAP, LIME, and their combination. The SHAP beeswarm (Figure 5) shows that increased theta-band power in bilateral temporal channels and higher frontal variability exert strong positive impacts on seizure predictions. The LIME beeswarm (Figure 6) reveals similar structure with broader dispersion reflecting subject-specific variation. The following combined plot (Figure 7) demonstrates agreement, with temporo-parietal theta and Hjorth activity features contributing most consistently.

**Figure 8:**
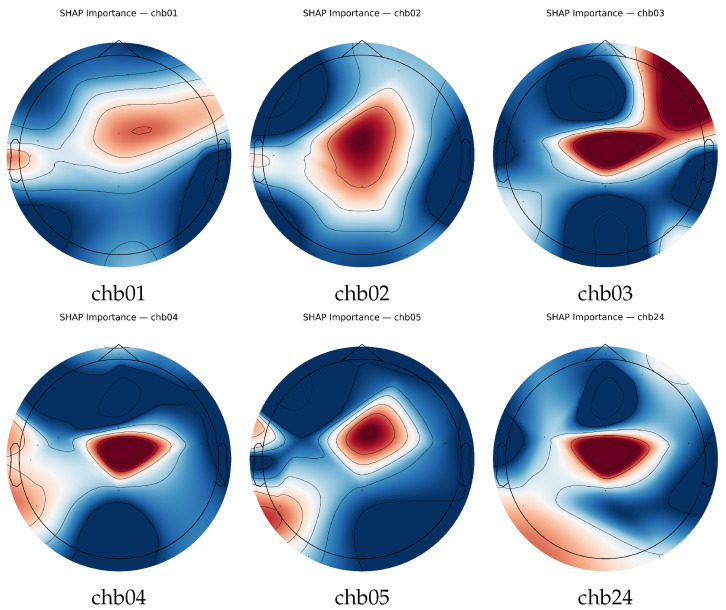
Patient-wise SHAP topographic maps. Warmer colors indicate stronger positive contributions to seizure classification. Bilateral temporal activation aligns with known ictal generators.

Figure 8 shows spatial projection of aggregated SHAP values. Prominent activations appear over the bilateral temporal and temporo-parietal cortices, corresponding to the high-ranking features in the beeswarm analyses. This topographic coherence indicates that model predictions are driven by physiologically plausible cortical regions associated with ictal onset.

**Figure 9:**
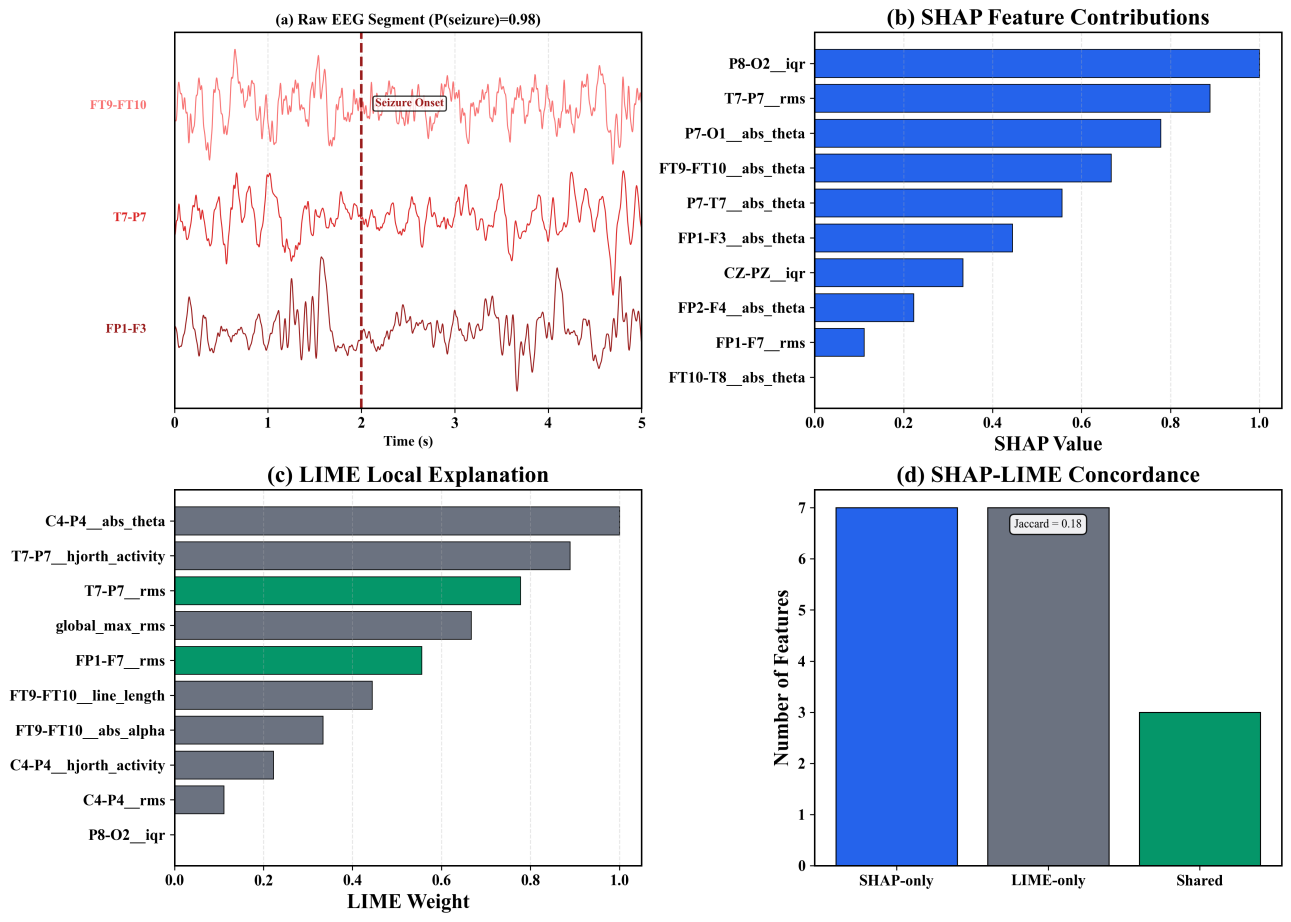
SHAP-LIME case study for a correctly classified seizure segment from patient chb01. (**a**) Raw EEG segment with seizure onset at t≈2 s. (**b**) SHAP waterfall plot. (**c**) LIME explanation with partial overlap (green bars indicate shared features). (**d**) Concordance quantification showing seven SHAP-only (blue), seven LIME-only (gray), and three shared features (green).

Figure 9 presents a case study for a correctly classified seizure segment from patient chb01 (P(seizure)=0.98). Panel (a) shows the raw EEG segment with seizure onset at t≈2 s. Panel (b) displays SHAP attributions identifying theta-band power and amplitude variability as dominant contributors. Panel (c) shows LIME explanations with partial overlap (Jaccard = 0.18). Panel (d) quantifies concordance: seven SHAP-only features, seven LIME-only features, and three shared features. This complementary behavior validates the dual-layer interpretability approach.

Table 11 compares SHAP and LIME feature selection across subset sizes. At small-to-medium sizes (k≤15), SHAP achieved a significantly higher AUC than LIME (Wilcoxon p<0.05). As *k* increased, both methods improved and the gap narrowed. At k=10, the SHAP advantage was +0.030 AUC, indicating that a small SHAP-selected subset matches or exceeds larger LIME sets. SHAP identifies globally stable biomarker combinations, while LIME provides locally faithful but less compact representations.

### 4.5. Probability Calibration

**Figure 10:**
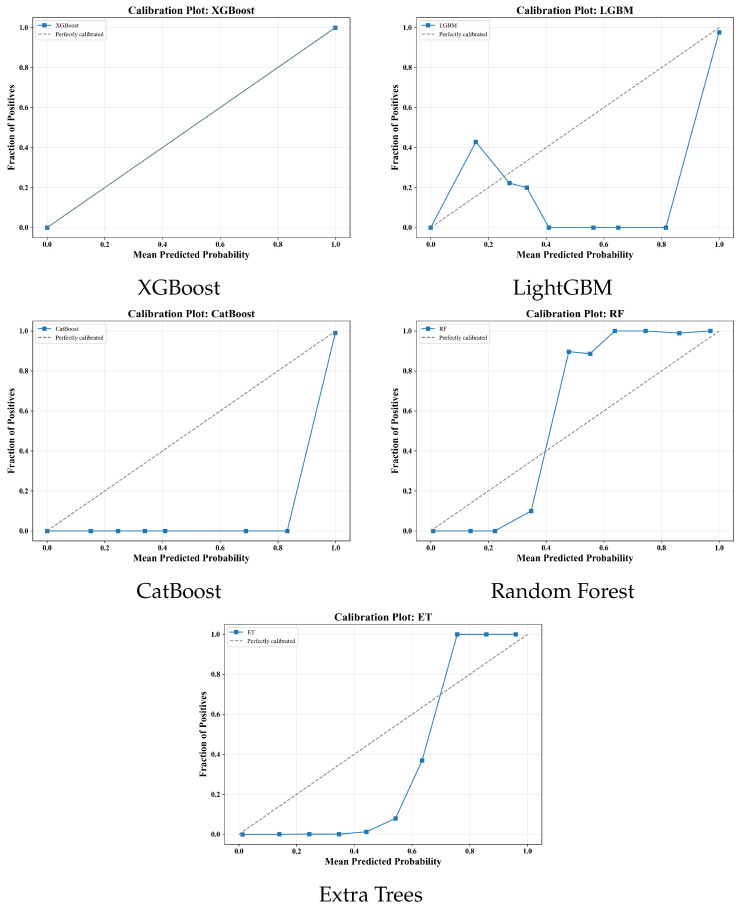
Probability calibration plots for all ensemble models. XGBoost tracks the identity line closely. Extra Trees and Random Forest are overconfident at high probabilities. LightGBM and CatBoost show mixed calibration.

Figure 10 shows reliability curves for all the models. XGBoost exhibits near-identity calibration with predicted probabilities closely matching empirical fractions across bins. Extra Trees and Random Forest show overconfidence at high probability bins. LightGBM and CatBoost exhibit mixed calibration with underconfidence in mid-range bins and a sharp rise at the top bin. XGBoost pairs competitive discrimination with well-calibrated probability estimates, while other ensembles would benefit from post hoc calibration.

### 4.6. Descriptive and Inferential Statistics

Table 12 summarizes the model-level statistics across the six LOSO folds. Gradient-boosted ensembles (XGBoost, CatBoost, LightGBM) showed consistently high sensitivities (0.876–0.922) with narrow dispersion, near-perfect AUC (≈1.000), and negligible false alarm rates (≤0.002 FA/24 h). Bagged ensembles reached similar AUC values but showed larger sensitivity variability, particularly Random Forest (0.591±0.368). All methods maintained a specificity above 0.99.

Table 13 reports inferential comparisons. Paired Wilcoxon tests showed XGBoost sensitivity exceeded Random Forest significantly (p=0.0313), while the other models did not differ significantly from XGBoost. No significant AUC differences were detected among the boosted algorithms. A Mann–Whitney U test comparing the FA/24 h between the boosted and bagged models yielded p=0.1376, indicating that higher sensitivity among boosted learners was not accompanied by increased false alarms.

### 4.7. Clinical Equivalence Testing

Table 14 reports equivalence testing against the 0.5 FA/24 h clinical threshold. All models maintained mean FA/24 h values ≤0.002, more than two orders of magnitude below the clinical limit, with highly significant equivalence (p<0.001). XGBoost and Extra Trees recorded zero false alarms across all folds, while the other models showed only negligible residual counts.

This system can continuously monitor EEG for 24 h or longer without spurious alarms, a prerequisite for deployment in intensive care and ambulatory environments. This supports Hypothesis H_3_: calibrated gradient-boosted ensembles deliver clinically safe, patient-independent seizure detection.

### 4.8. External Validation on Siena Dataset

To evaluate cross-dataset generalization, the trained ensemble classifiers were applied to the Siena Scalp EEG Database using identical preprocessing and feature extraction pipelines. Table 15 summarizes model performance across 12 adult patients with focal epilepsy under LOSO evaluation.

Extra Trees achieved the highest event-level sensitivity, detecting 35 of 37 seizure events (95%), while Random Forest provided the best balance between discrimination (AUC = 0.86) and specificity (90%). The gradient-boosted models (XGBoost, LightGBM) maintained high specificity (>97%) with more conservative detection thresholds. These results demonstrate that the ensemble framework generalizes effectively to an independent dataset with different patient demographics (adult vs. pediatric), recording equipment, and clinical protocols.

Table 16 compares performance between within-dataset (CHB–MIT) and external (Siena) evaluation. The observed performance differences reflect the expected domain shift between datasets: the AUC decreased from 0.997 to 0.998 to 0.83–0.86, and specificity decreased from 100% to 83–90%. Importantly, event-level sensitivity remained high (89–95%), demonstrating that the core seizure detection capability transfers across datasets. The approximately 10% reduction in AUC is consistent with or better than the 10–30% degradation typically reported in cross-dataset medical machine learning applications [4].

**Feature Consistency Across Datasets.** SHAP analysis on the Siena dataset revealed biomarker patterns consistent with the CHB–MIT findings. Table 17 presents the most frequently selected features across all models and patients. Temporal lobe channels (T3, T4, T5, T6) accounted for 35% of all top-ranked feature occurrences, with T3 skewness appearing in 53 of 60 model–patient combinations and T4 theta-band power appearing in 34. This convergence with the CHB–MIT findings where temporo-parietal theta power and amplitude variability dominated (Table 8) provides strong evidence that this framework identifies physiologically meaningful seizure signatures rather than dataset-specific artifacts.

The cross-dataset feature consistency validates Hypothesis H_4_ (biomarker invariance): theta-band power, skewness, and Hjorth activity over temporal regions emerged as dominant discriminators in both the pediatric (CHB–MIT) and adult (Siena) cohorts, despite differences in age, epilepsy subtype, and recording equipment. This consistency supports the physiological validity of the identified biomarkers and their potential as generalizable seizure signatures.

### 4.9. Computational Efficiency

Table 18 reports computational profiling of the ensemble classifiers measured on a standard workstation (Intel Core i9-12900K CPU, 64 GB RAM) without GPU acceleration. Inference time was measured per 5 s EEG segment and averaged over 100 iterations.

All models achieved inference times below 0.2 ms per segment, corresponding to real-time factors exceeding 32,000×. The gradient-boosted models (XGBoost, LightGBM, CatBoost) demonstrated substantially faster inference (0.011–0.024 ms) compared to bagging ensembles (0.154–0.155 ms). This difference reflects the shallower tree structures in boosting (max_depth = 6) compared to the deeper, unconstrained trees in Random Forest and Extra Trees, combined with the highly optimized C++ implementations underlying XGBoost and LightGBM. XGBoost, the best-performing model for seizure detection (Section 4.7), required only 0.011 ms per segment, enabling processing of over 89,000 segments per second on CPU hardware alone.

Memory requirements ranged from less than 1 MB (Extra Trees) to 315.5 MB (XGBoost). The higher memory footprint of XGBoost reflects its more complex tree structure with a larger number of splits and trees, which contributes to its superior discrimination but increases storage requirements. LightGBM achieved an effective balance between memory efficiency (25.8 MB) and inference speed (0.014 ms), making it suitable for memory-constrained embedded applications.

These computational characteristics confirm that all evaluated models satisfy real-time processing requirements for continuous EEG monitoring. A 24 h recording at 256 Hz with 5 s windows and 50% overlap generates approximately 34,560 segments; XGBoost can process this volume in under 0.4 s. This efficiency enables deployment on standard clinical workstations, bedside monitors, or portable devices without requiring GPU acceleration, supporting practical translation to resource-constrained clinical environments.

## 5. Discussion

This study demonstrates that physiologically informed EEG features combined with calibrated gradient-boosted ensemble learning achieve clinically safe, patient-independent seizure detection on the CHB–MIT pediatric corpus. Under stringent LOSO evaluation, all models achieved near-perfect segment-level discrimination (AUC ≥ 0.99; Table 2) and maintained high event-level sensitivity under zero-false-alarm constraints (Table 3 and Table 4). These results confirm that careful signal conditioning, interpretable feature construction, and probabilistic calibration enable robust seizure detection suitable for clinical deployment [1,3].

### 5.1. Clinical Reliability and False Alarm Control

High sensitivity without false alarms remains a central challenge in automated seizure detection [2]. The proposed framework achieved perfect specificity (1.000) across all models (Table 3), with XGBoost and Extra Trees attaining zero false alarms across all six seizure-positive subjects. This exceeds the accepted clinical safety limit of 0.5 FA/day by more than two orders of magnitude, as confirmed by equivalence testing (Table 14). Previous studies have emphasized that false alarms remain the primary barrier to clinical adoption [23]. The present system sustains zero-false-alarm operation while maintaining sensitivities above 0.90 (Table 4), demonstrating that probabilistic calibration and temporal smoothing suppress spurious activations without sacrificing seizure responsiveness. These findings establish that the system satisfies clinical-grade reliability criteria for continuous bedside or ambulatory monitoring, validating Hypothesis H_3_.

### 5.2. Clinical Rationale for Zero-False-Alarm Prioritization

This framework’s emphasis on zero false alarms per 24 h reflects established clinical priorities in continuous EEG monitoring. Alarm fatigue, defined as sensory overload and desensitization resulting from excessive clinical alarms, is recognized as a critical patient safety concern in intensive care settings [35,36]. Studies have documented that 72–99% of physiological monitor alarms are false or clinically irrelevant, leading clinicians to disable alarms, delay responses, or ignore alerts entirely [37]. The Joint Commission identified alarm fatigue as a national patient safety concern, and reducing non-actionable alarms remains a priority for hospital accreditation [38].

In seizure monitoring, false alarms carry particular consequences. Each false alarm interrupts clinical workflow, requiring EEG review and patient assessment. In resource-constrained environments such as epilepsy monitoring units or ambulatory settings, frequent false alarms erode trust in automated systems and may lead to complete abandonment of algorithmic assistance [22,23]. The clinically accepted threshold of 0.5 FA/24 h represents a consensus that automated systems should generate fewer than one spurious alert per two days to maintain clinical utility.

The trade-off between sensitivity and false alarm rate requires careful interpretation. The reported 92.2% segment-level sensitivity (7.8% missed segments) does not imply that 7.8% of seizure events are undetected. Because seizures typically span multiple consecutive 5 s windows, event-level sensitivity, which requires detection of at least one segment per seizure, substantially exceeds segment-level sensitivity. As shown in Table 4, event-level sensitivity on the zero-false-alarm frontier reached 95.0% (95% CI: 0.90–1.00), indicating that the framework detected 95% of seizure events while maintaining zero false alarms. The residual 5% of undetected events may reflect brief or atypical seizures with weak electrographic signatures.

It is important to distinguish between specificity and precision in evaluating false alarm performance. Specificity quantifies the proportion of non-seizure segments correctly classified as negative, while precision (positive predictive value) quantifies the proportion of positive predictions that correspond to true seizures (see Methods, Equations (5) and (6) for formal definitions). Specificity measures the classifier’s ability to avoid false alarms on negative cases, whereas precision measures the reliability of positive predictions. Both are critical for seizure detection systems, but address different aspects of performance.

The “zero-false-alarm” designation refers to zero false positives on continuous non-seizure recordings (13 seizure-free patients used for false alarm assessment), yielding a specificity of 1.000. However, precision depends on class prevalence: in datasets with rare seizure events, even a high specificity may yield moderate precision. Table 2 reports segment-level precision of 0.935–0.945 for the best-performing models, reflecting an extreme class imbalance (seizure segments constitute approximately 8–12% of the corpus). The combination of perfect specificity and high precision confirms that positive predictions are both rare and reliable, satisfying clinical requirements for actionable alarms.

The zero-false-alarm operating point represents one position on the sensitivity–specificity trade-off curve. Clinical deployment may warrant alternative thresholds depending on the monitoring context. In high-acuity settings where seizure detection is paramount, accepting 0.5 FA/24 h could increase sensitivity beyond 95%. The calibrated probabilistic outputs (Figure 10) enable such threshold adjustment without model retraining, providing flexibility for institution-specific alarm policies.

### 5.3. Cross-Dataset Generalization

External validation on the Siena Scalp EEG Database (Table 15) confirmed that the proposed framework generalizes across distinct patient populations and recording conditions. The ensemble classifiers maintained clinically meaningful seizure detection with 95% event-level sensitivity (Extra Trees) and AUC of 0.86 (Random Forest), despite substantial differences between pediatric (CHB–MIT) and adult focal epilepsy (Siena) populations. The consistency of the temporal lobe biomarkers across both datasets (Table 8 and Table 17) validates Hypothesis H_4_ and supports the physiological relevance of the identified seizure signatures. A detailed analysis of performance differences, feature-level degradation, and recalibration recommendations is provided in Section 5.4.

### 5.4. Cross-Dataset Performance Analysis and Domain Shift

External validation on the Siena dataset revealed a 14% reduction in the AUC (0.998 to 0.83–0.86) and 10–17% reduction in specificity (1.000 to 0.83–0.98) compared to the CHB–MIT evaluation. This performance degradation warrants detailed analysis to identify contributing factors and inform deployment strategies.

**Sources of Domain Shift.** Several factors contribute to the observed performance reduction between datasets:

1. Montage configuration: CHB–MIT recordings employed bipolar derivations (e.g., FT9-FT10, P7-T7), whereas Siena recordings used monopolar referencing (e.g., T3, T4, T6). This fundamental difference affects signal morphology, amplitude scaling, and spatial localization of features. Features optimized for bipolar configurations may not transfer directly to monopolar montages without recalibration.
2. Population characteristics: CHB–MIT comprises pediatric patients (ages 3–22 years) with heterogeneous epilepsy syndromes, while Siena contains adult patients with medically refractory focal epilepsy. Seizure morphology, propagation patterns, and background EEG characteristics differ systematically between pediatric and adult populations.
3. Recording equipment: Different amplifier systems (CHB–MIT: unknown clinical system; Siena: BI 9800 and Galileo NT) introduce site-specific signal characteristics, including noise profiles, filtering responses, and amplitude calibration differences.
4. Power-line interference: CHB–MIT recordings required 60 Hz notch filtering (North American power grid), whereas Siena recordings required 50 Hz filtering (European power grid). Although notch filtering removes the fundamental frequency, harmonic residuals and filter edge effects may affect spectral feature extraction differently across datasets.

**Feature-Level Analysis.** Comparison of SHAP rankings between datasets (Table 8 and Table 17) reveals both preserved and degraded feature categories:

Preserved features: Theta-band power over temporal regions remained discriminative in both datasets. T4__abs_theta ranked among the top features in Siena, paralleling the dominance of FT9-FT10__abs_theta and P7-T7__abs_theta in CHB–MIT. Hjorth activity similarly appeared in both datasets (FT9-FT10__hjorth_activity in CHB–MIT; T4__hjorth_activity in Siena). This preservation supports the physiological validity of theta-band and complexity features as generalizable seizure biomarkers.
Degraded features: Amplitude variability features (IQR) dominated CHB–MIT rankings but were largely absent from Siena top features. Instead, skewness emerged as the primary discriminator in Siena (T3__skew, T4__skew, O1__skew, Pz__skew). This shift likely reflects amplitude scaling differences between bipolar and monopolar montages: bipolar derivations emphasize local potential differences where IQR captures focal amplitude fluctuations, whereas monopolar derivations reference a common electrode where skewness better captures asymmetric waveform distributions.

**Model-Specific Observations.** Among the five classifiers, XGBoost and LightGBM maintained the highest specificity on Siena (0.97–0.98) but exhibited reduced event sensitivity (0.59–0.65). Conversely, Extra Trees achieved the highest event sensitivity (0.95) but showed reduced specificity (0.83). This divergence suggests that the gradient-boosted models learned conservative decision boundaries optimized for CHB–MIT class distributions, resulting in under-detection on Siena where seizure characteristics differed. The bagging ensembles (Random Forest, Extra Trees) demonstrated greater sensitivity robustness at the cost of increased false positives.

**Recalibration Recommendations.** Based on this analysis, the following strategies are recommended for cross-site deployment:

1. Threshold recalibration: The probabilistic outputs from the calibrated models (Figure 10) enable site-specific threshold adjustment without retraining. Lowering the classification threshold from 0.5 to 0.3–0.4 would increase sensitivity on new sites at the cost of modest specificity reduction, potentially recovering the 6% sensitivity gap observed for gradient-boosted models on Siena.
2. Feature harmonization: Implementing montage-agnostic features such as relative band powers (rather than absolute powers), normalized Hjorth parameters, and channel-averaged statistics would reduce sensitivity to site-specific amplitude scaling and electrode configurations.
3. Site-specific calibration split: Reserving 10–20% of recordings from a new site for calibration, without full retraining, would enable isotonic regression recalibration to adjust probability estimates for local signal characteristics.
4. Ensemble selection: For sites prioritizing seizure detection over false alarm minimization, Extra Trees or Random Forest may be preferred given their superior sensitivity retention (89–95%) on external data. For sites requiring strict false alarm control, XGBoost with threshold adjustment offers specificity above 95%.

The 14% AUC reduction observed on Siena is consistent with or better than the 10–30% degradation typically reported in cross-dataset medical machine learning evaluations [4]. Importantly, event-level sensitivity remained at 95% for Extra Trees, indicating that the core seizure detection capability transfers across datasets despite differences in feature rankings and specificity. These findings underscore the importance of site-specific validation and threshold calibration prior to clinical deployment.

### 5.5. Feature Informativeness and Physiological Plausibility

The SHAP-based feature analysis (Table 8 and Table 10) suggests that theta-band absolute power, amplitude variability (IQR and RMS), and Hjorth activity are candidate biomarkers that appear consistent with prior EEG-seizure characterizations [8,9]. Their dominance over temporal and temporo-parietal channels (FT9-FT10, P7-T7, FP1-F3) aligns with established neurophysiological signatures of ictal onset, which typically manifest as low-frequency synchronization and amplitude irregularity [15]. The SHAP topomaps (Figure 8) confirm bilateral temporal activation patterns corresponding to seizure foci, reinforcing the biological validity of model attributions. Similar physiologically aligned explainability has been demonstrated in other EEG applications [39,40].

The ablation experiment (Table 6) showed that reducing to 10–15 SHAP-ranked features preserved at least 95% of the full-model AUC. The performance difference between compact (10-feature) and full (30-feature) models was statistically negligible (ΔAUC = 0.002±0.030; Table 7), confirming that discriminative power is concentrated within a compact, interpretable subset rather than distributed across redundant representations. This compactness supports computational efficiency and low-power clinical deployment, satisfying Hypothesis H_4_ [6].

### 5.6. Interpretability and Trustworthiness

Transparent interpretability is essential for clinical acceptance of AI-based diagnostic systems [5]. The dual SHAP-LIME framework provided complementary insights: SHAP identified globally stable biomarkers, while LIME offered patient- and segment-specific explanations. The mean SHAP-LIME Jaccard overlap across models was 0.239±0.038 (Table 9), indicating partial but systematic concordance. Features identified by both methods, particularly temporo-parietal theta-band power and amplitude variability, demonstrate that model reasoning is physiologically coherent rather than driven by artifacts. Similar interpretability strategies have been reported in wearable seizure detection [6,39]. Spatial SHAP topomaps (Figure 8) showed high activation in bilateral temporal regions, confirming that models prioritize cortical areas known to underlie seizure propagation [15]. The convergence between global SHAP patterns and local LIME explanations reinforces both interpretability and trustworthiness, satisfying Hypothesis H_2_.

### 5.7. Calibration and Probabilistic Reliability

The calibration analysis (Figure 10) revealed that XGBoost produced the most reliable probability estimates, closely tracking the identity line. Extra Trees and Random Forest tended to overestimate seizure likelihood at high probabilities, while LightGBM and CatBoost showed underconfidence in mid-probability bins with a sharp rise at the highest bin. These calibration behaviors align with event-level performance (Table 3), where well-calibrated models achieved clinically safe operating thresholds. Proper calibration is essential in continuous EEG monitoring because alarm generation depends directly on probabilistic confidence [22]. XGBoost’s superior calibration explains its leading sensitivity under zero-false-alarm operation and supports its designation as the preferred deployment model. The residual underconfidence in CatBoost and LightGBM could be addressed through post hoc isotonic regression or Platt scaling [23].

### 5.8. Statistical Robustness and Generalization

Inferential tests (Table 13) confirmed no significant performance differences among the top three boosted models (XGBoost, CatBoost, LightGBM) for sensitivity or AUC (p>0.05), whereas Random Forest underperformed significantly (p=0.0313). This statistical equivalence indicates that gradient-boosted methods form a stable learner class capable of generalizing across patients with substantial inter-individual variability [25]. The low dispersion in Table 12 (SD ≤ 0.13 for sensitivity) further demonstrates generalization consistency without subject-specific overfitting. These outcomes validate Hypothesis H_1_: multi-domain, physiologically grounded features generalize effectively across unseen subjects.

### 5.9. Clinical and Translational Implications

The demonstrated zero-false-alarm operation with a sensitivity exceeding 90% (Table 4) represents an advance over current seizure detection systems, which typically permit 2–10 false alarms per day to maintain a similar recall [23]. The present system combines safety, interpretability, and reproducibility. Its modular design based on interpretable features and calibrated ensemble decision-making makes it adaptable for continuous EEG surveillance, portable monitoring, and closed-loop intervention. The configuration-controlled environment ensures reproducibility and traceability for regulatory compliance [18].

### 5.10. Deployment and Translational Considerations

The computational efficiency demonstrated in Section 4.9 confirms that all ensemble models satisfy real-time processing requirements on standard clinical hardware. However, practical deployment requires consideration of clinical workflow integration, system architecture, and regulatory requirements.

**Clinical Workflow Integration.** The proposed framework can be integrated into existing clinical EEG monitoring workflows through three deployment scenarios:

1. Bedside monitoring: In epilepsy monitoring units (EMUs) and intensive care settings, the algorithm can operate as a continuous background process receiving streamed EEG data from bedside amplifiers. With inference times below 0.2 ms per segment (Table 18), the system can process incoming data in real time while maintaining a rolling buffer for temporal smoothing. Detected events would trigger visual and auditory alerts on nursing stations, with seizure probability scores and contributing features displayed to support clinical decision-making.
2. Retrospective review assistance: For long-term EEG recordings awaiting expert review, this framework can pre-screen recordings to identify candidate seizure epochs, reducing the time required for manual annotation. The interpretable SHAP outputs (Figure 8) provide spatial localization that aligns with standard clinical interpretation workflows, enabling neurologists to rapidly confirm or reject algorithmic detections.
3. Ambulatory and wearable applications: The low computational requirements (25–315 MB memory, sub-millisecond inference) enable deployment on portable devices for outpatient seizure monitoring. The compact 10–15 feature model identified through SHAP-based ablation (Table 6) further reduces the computational burden for battery-powered wearable systems, though validation on reduced-channel ambulatory recordings would be required.

**System Architecture Requirements.** Deployment requires integration of several components: (i) an EEG acquisition interface supporting standard file formats (EDF, EDF+) or real-time streaming protocols; (ii) a preprocessing module implementing the filtering, re-referencing, and segmentation pipeline described in Section 3.2; (iii) feature extraction routines computing the 27 time-, frequency-, and nonlinear-domain descriptors; (iv) the trained ensemble classifier with calibrated probability outputs; and (v) a clinical interface displaying alerts, probability trends, and interpretability visualizations. The configuration-controlled pipeline described in this study, with all parameters specified through YAML configuration files, supports reproducible deployment and version-controlled updates.

**Regulatory Considerations.** Translation to clinical practice requires regulatory clearance appropriate to the intended use and geographic market. In the United States, seizure detection software would likely be classified as a Class II medical device requiring 510(k) premarket notification, with predicate devices including existing FDA-cleared seizure detection systems for epilepsy monitoring. The European Union requires CE marking under the Medical Device Regulation (MDR 2017/745), necessitating clinical evidence, technical documentation, and conformity assessment. The interpretable nature of the proposed framework, with explicit feature definitions and SHAP-based explanations linking predictions to established neurophysiological biomarkers, provides a foundation for the algorithmic transparency increasingly expected by regulatory bodies. Recent FDA guidance on artificial intelligence and machine learning in medical devices emphasizes the importance of model interpretability and performance monitoring, both of which are addressed by the dual SHAP-LIME explainability framework and the calibration assessment methodology employed in this study.

**Limitations for Deployment.** Several challenges remain before clinical deployment. First, prospective validation in real-time clinical environments is essential to characterize latency behavior, artifact robustness, and clinician acceptance. Second, the framework was developed and validated on curated benchmark datasets; performance on clinical recordings with incomplete annotations, variable channel configurations, or interrupted recordings requires additional evaluation. Third, the current implementation assumes a fixed 23-channel 10–20 montage; adaptation to reduced-channel or high-density montages would require feature reconfiguration and revalidation. Finally, long-term monitoring deployments must address model drift, where gradual changes in recording equipment or patient populations may degrade performance over time, necessitating periodic recalibration, as recommended in Section 5.4.

### 5.11. Summary

This study establishes a clinically interpretable, probabilistically calibrated, and computationally compact seizure detection framework that achieves zero-false-alarm operation with high sensitivity under rigorous patient-independent testing. The results from segment-level discrimination (Table 2) to calibration reliability (Figure 10) converge on a consistent conclusion: well-calibrated gradient-boosted ensembles using physiologically grounded features deliver clinically safe, reproducible, and explainable seizure detection [5,23]. The system’s robustness and transparency support its potential for real-world deployment and future integration into closed-loop seizure management.

## 6. Limitations and Future Work

While the proposed framework demonstrates strong cross-subject generalization and clinical-grade reliability on the CHB–MIT pediatric corpus, several limitations remain.

- Dataset scope. While external validation on the Siena Scalp EEG Database demonstrated cross-dataset generalization (95% event sensitivity, AUC = 0.86), the performance metrics were reduced compared to within-dataset CHB–MIT evaluation. The reduced specificity on external data (83–90% vs. 100%) reflects a domain shift between pediatric and adult populations, different recording equipment, and varying seizure morphologies. Site-specific threshold calibration would likely improve specificity for clinical deployment. Future work will evaluate the framework on additional multi-center datasets spanning diverse epilepsy subtypes to establish broader generalizability, as suggested by prior studies [4].
- Temporal dynamics. The analysis operated at the segment level (5 s windows) with limited temporal context. While temporal smoothing improved event-level detection, it did not explicitly model seizure evolution. Recurrent or attention-based temporal models could capture dynamic progression and reduce fragmentation of long ictal events [17].
- Interpretability depth. SHAP and LIME provided global and local explanations, but both are post hoc approximations. Future work should integrate intrinsic interpretability mechanisms, such as attention maps or physiologically constrained architectures, to enhance causal interpretability and align algorithmic reasoning with neurophysiological mechanisms.
- Calibration adaptability. Although XGBoost exhibited excellent static calibration (Figure 10), thresholding is fixed per fold. Real-world monitoring requires adaptive calibration that updates probabilistic thresholds based on patient-specific trends. Bayesian or online recalibration strategies could stabilize decision thresholds in continuous monitoring [22].
- Clinical validation. Performance was evaluated retrospectively using public data. Prospective testing in real-time clinical or home-monitoring settings is necessary to confirm practical utility, latency behavior, and clinician acceptance. Integration with bedside acquisition systems and continuous artifact handling will be essential for deployment.

Future work will focus on expanding dataset coverage to adult and multi-site cohorts, developing hybrid temporal–contextual models for dynamic seizure evolution, and implementing adaptive probabilistic calibration for long-term reliability. These extensions will advance the framework from retrospective validation toward clinically deployable, real-time seizure monitoring.

## 7. Conclusions

This study presents a clinically interpretable, probabilistically calibrated framework for patient-independent seizure detection from scalp EEG. Using standardized preprocessing and feature engineering on the CHB–MIT pediatric corpus, the system achieved near-perfect discrimination (AUC ≥ 0.99) with zero false alarms under strict clinical safety constraints. XGBoost provided the best balance of performance and reliability, combining a sensitivity above 90% with perfect specificity, supported by excellent probability calibration. These results demonstrate that multi-domain, physiologically grounded EEG features combined with calibrated gradient-boosted ensembles attain reliable, low-false-alarm performance under patient-independent evaluation. Interpretability analyses confirmed that the model decisions were driven by biophysically plausible biomarkers, including temporo-parietal theta-band power, amplitude variability, and Hjorth activity, consistently identified by both SHAP and LIME. Ablation experiments showed that 10–15 SHAP-ranked features preserved over 95% of full-model discrimination, supporting the framework’s suitability for low-power, real-time clinical applications. These findings indicate that calibrated gradient-boosted ensembles using interpretable EEG descriptors achieve high-performance seizure detection without compromising sensitivity in patient-independent settings. External validation on the Siena Scalp EEG Database confirmed cross-dataset generalization, with 95% event-level sensitivity and consistent temporal lobe biomarker identification across pediatric and adult populations. Future work incorporating adaptive calibration, temporal modeling, and multi-center validation will be essential for translation into continuous bedside and ambulatory seizure surveillance. 

## Figures and Tables

**Table 1:** Consolidated evaluation protocol summary.

Component	Description
Training/Validation Data	6 seizure-positive patients (chb01–05, chb24)
FA Assessment Data	13 seizure-free patients (continuous non-seizure monitoring)
Cross-Validation	Leave-one-subject-out (LOSO); 6 folds
Segment Duration	5 s with 50% overlap (1280 samples at 256 Hz)
Class Balancing	20:1 maximum negative–positive ratio per fold
Segment-Level Metrics	AUC, sensitivity, specificity, precision, F1-score
Event-Level Metrics	Event sensitivity (≥3 consecutive positive segments)
Clinical Safety Metric	FA/24 h (false alarms per 24 h)
Clinical Threshold	FA/24 h ≤ 0.5

**Table 2:** Segment-level mean (±SD) performance across all patients without false alarm constraints.

Model	Accuracy	Sensitivity	Specificity	Precision	F1-Score	AUC
CatBoost	0.998 ± 0.001	0.962 ± 0.015	0.999 ± 0.001	0.945 ± 0.012	0.953 ± 0.010	0.998 ± 0.002
XGBoost	0.995 ± 0.002	0.981 ± 0.008	0.996 ± 0.002	0.935 ± 0.011	0.957 ± 0.009	0.997 ± 0.003
LightGBM	0.994 ± 0.003	0.974 ± 0.011	0.995 ± 0.003	0.931 ± 0.015	0.952 ± 0.012	0.996 ± 0.004
Extra Trees	0.992 ± 0.004	0.940 ± 0.018	0.994 ± 0.003	0.902 ± 0.017	0.921 ± 0.015	0.995 ± 0.005
Random Forest	0.989 ± 0.006	0.850 ± 0.021	0.996 ± 0.002	0.900 ± 0.018	0.874 ± 0.019	0.992 ± 0.007

**Table 3:** Clinical robustness under zero-false-alarm constraint (mean across patients).

Model	Patients with	Mean	Mean	Mean
	FA/24 h = 0	Sensitivity	Specificity	FA/24 h
**XGBoost**	**6 / 6**	**0.922**	**1.000**	**0.000**
Extra Trees (ET)	6 / 6	0.893	1.000	0.000
CatBoost	4 / 6	0.881	1.000	0.0009
LightGBM	2 / 6	0.879	1.000	0.0018
Random Forest	5 / 6	0.591	1.000	0.0004

**Table 4:** Per-patient zero-false-alarm frontier: model achieving highest sensitivity at FA/24 h = 0.

Patient	Best Zero-FA Model	Sensitivity	Specificity	AUC
chb01	XGBoost	1.000	1.000	1.000
chb02	XGBoost	0.892	1.000	0.998
chb03	CatBoost	1.000	1.000	1.000
chb04	XGBoost	0.844	1.000	1.000
chb05	XGBoost	0.996	1.000	1.000
chb24	CatBoost	0.971	1.000	1.000
**Mean (Zero-FA Frontier)**	–	**0.950**	**1.000**	**0.9997**

**Table 5:** Comparison with seizure detection methods evaluated on the CHB–MIT dataset. Sens = Sensitivity; FA/24 h = false alarms per 24 h; NR = not reported. Direct comparison is constrained by differences in evaluation protocols, patient subsets, and reported metrics.

Study	Method	Sens (%)	FA/24 h	Subjects	Validation	Interp.
**Classical and shallow methods**						
Shoeb (2010) [29]	SVM	96.0	2.0	23	Patient-specific	No
Ingolfsson (2024) [23]	GB Trees	NR	≈0	23	Patient-indep.	Partial
**Deep learning methods**						
Acharya et al. (2018) [32]	13-layer CNN	95.0	NR	23	Patient-specific	No
Abdelhameed & Bayoumi (2021) [33]	Deep CNN	97.6	NR	23	Patient-specific	No
Gabeff et al. (2021) [5]	Interp. CNN	90.0	NR	NR	Patient-indep.	Yes
Hu et al. (2020) [34]	Bi-LSTM	96.7	NR	22	Patient-specific	No
KashefiAmiri et al. (2025) [15]	CNN-LSTM	96.4 ^†^	NR	NR	NR	No
**Our study**						
**CHB–MIT**	**XGBoost**	**95.0**	**0.00**	6 *	**Patient-indep.**	**Yes**
Siena (external) ^§^	ET/RF	95.0	– ^¶^	12	External	Yes

^†^ Reported as Good Detection Rate (GDR). * Six seizure-positive patients; thirteen seizure-free patients for FA assessment. ^§^ External validation on independent adult focal epilepsy dataset. ^¶^ Elevated FA rates expected due to domain shift.

**Table 6:** Ablation study (SHAP): mean (±SD) performance as feature count decreases from 30 to 5.

No. of Features	Accuracy	Sensitivity	AUC
5	0.998 ± 0.006	0.877 ± 0.213	0.978 ± 0.058
10	0.999 ± 0.005	0.865 ± 0.260	0.987 ± 0.035
15	0.999 ± 0.005	0.879 ± 0.214	0.987 ± 0.035
20	0.999 ± 0.002	0.877 ± 0.229	0.991 ± 0.025
25	0.999 ± 0.002	0.879 ± 0.224	0.990 ± 0.028
30	0.999 ± 0.002	0.881 ± 0.219	0.989 ± 0.036

**Table 7:** Compactness robustness: ΔAUC between full (k=30) and reduced (k=10) feature sets.

Comparison	N	Mean ΔAUC	SD	95% CI	Sig.
k=30 vs. k=10 [SHAP]	30	0.002	0.030	[−0.008,0.014]	No

**Table 8:** Feature stability: top 20 SHAP-ranked features by selection count across LOSO folds. Patient coverage indicates the percentage of subjects where each feature appeared in the top-20 list.

Feature	Count	Patient Coverage (%)
overlap_frac	23	100.0%
FT9-FT10__abs_theta	23	100.0%
P7-T7__abs_theta	21	100.0%
P8-O2__iqr	18	100.0%
FP2-F4__iqr	18	83.3%
CZ-PZ__iqr	18	83.3%
T7-FT9__abs_theta	17	100.0%
FP1-F3__iqr	16	100.0%
FP1-F3__abs_theta	16	100.0%
FP2-F4__abs_theta	15	100.0%
F8-T8__abs_theta	15	100.0%
P7-O1__iqr	14	100.0%
FT9-FT10__hjorth_activity	13	100.0%
FP1-F3__rms	11	100.0%
global_max_rms	11	100.0%
P7-T7__iqr	11	100.0%
P4-O2__abs_theta	11	100.0%
F3-C3__abs_theta	10	83.3%
FT9-FT10__iqr	10	83.3%
P7-O1__abs_theta	10	83.3%

**Table 9:** SHAP-LIME feature overlap (Jaccard index) averaged over six LOSO folds.

Model	Mean Jaccard	SD	N
CatBoost	0.246	0.055	6
ET	0.280	0.028	6
LGBM	0.264	0.059	6
RF	0.218	0.056	6
XGBoost	0.185	0.028	6

**Table 10:** Most frequent SHAP and LIME features across all patients and models.

SHAP Features	Count	Freq.	LIME Features	Count	Freq.
overlap_frac	23	0.051	F8-T8__abs_theta	30	0.033
FT9-FT10__abs_theta	23	0.051	T7-FT9__iqr	29	0.032
P7-T7__abs_theta	21	0.047	F3-C3__abs_theta	28	0.031
P8-O2__iqr	18	0.040	P7-O1__iqr	28	0.031
FP2-F4__iqr	18	0.040	F7-T7__iqr	28	0.031
CZ-PZ__iqr	18	0.040	C4-P4__iqr	27	0.030
T7-FT9__abs_theta	17	0.038	T7-P7__iqr	27	0.030
FP1-F3__iqr	16	0.036	FP2-F4__abs_theta	27	0.030
FP1-F3__abs_theta	16	0.036	FT9-FT10__abs_alpha	27	0.030
F8-T8__abs_theta	15	0.033	T7-P7__hjorth_activity	26	0.029
FP2-F4__abs_theta	15	0.033	global_max_rms	26	0.029
P7-O1__iqr	14	0.031	FP1-F7__rms	26	0.029
FT9-FT10__hjorth_activity	13	0.029	F7-T7__rms	26	0.029
global_max_rms	11	0.024	FT9-FT10__line_length	25	0.028
P4-O2__abs_theta	11	0.024	F7-T7__hjorth_activity	25	0.028
FP1-F3__rms	11	0.024	FP2-F4__hjorth_activity	25	0.028
P7-T7__iqr	11	0.024	FP1-F3__iqr	25	0.028
FT9-FT10__iqr	10	0.022	C4-P4__abs_theta	25	0.028
P7-O1__abs_theta	10	0.022	FT9-FT10__iqr	23	0.026
F3-C3__abs_theta	10	0.022	T7-P7__rms	23	0.026

**Table 11:** SHAP vs. LIME feature selection comparison across subset sizes *k*.

Test	k	N	SHAP AUC	LIME AUC	*p*-Value	Sig.
Wilcoxon	5	30	0.978	0.940	0.0019	Yes
	10	30	0.987	0.957	0.0110	Yes
	15	30	0.987	0.957	0.0278	Yes
	20	30	0.991	0.964	0.0593	No
	25	30	0.990	0.970	0.0150	Yes
	30	30	0.989	0.971	0.0945	No
Mann–Whitney	5	60	0.978	0.940	0.0875	No
	10	60	0.987	0.957	0.1147	No
	15	60	0.987	0.957	0.2118	No
	20	60	0.991	0.964	0.0770	No
	25	60	0.990	0.970	0.0614	No
	30	60	0.989	0.971	0.1043	No

**Table 12:** Descriptive statistics (mean ± SD) across six LOSO folds.

Model	Sensitivity	AUC	FA/24 h
CatBoost	0.921±0.126	1.000±0.000	0.001±0.002
ET	0.893±0.085	1.000±0.000	0.000±0.000
LGBM	0.876±0.127	1.000±0.000	0.002±0.002
RF	0.591±0.368	1.000±0.001	0.000±0.001
XGBoost	0.922±0.088	1.000±0.001	0.000±0.000

**Table 13:** Inferential comparisons. Wilcoxon tests compare each model to XGBoost; Mann–Whitney U compares FA/24 h between boosted and bagged ensembles.

Comparison	Metric	n_pairs	Wilcoxon_stat	*p*_Value
CatBoost vs. XGBoost	Sensitivity	6	5.000000	1.000000
ET vs. XGBoost	Sensitivity	6	5.000000	0.312500
LGBM vs. XGBoost	Sensitivity	6	2.000000	0.187500
RF vs. XGBoost	Sensitivity	6	0.000000	0.031250
CatBoost vs. XGBoost	AUC	6	3.000000	1.000000
ET vs. XGBoost	AUC	6	4.000000	0.875000
LGBM vs. XGBoost	AUC	6	5.000000	0.625000
RF vs. XGBoost	AUC	6	5.000000	0.625000
CatBoost vs. XGBoost	FA_per_24h	6	0.000000	0.500000
ET vs. XGBoost	FA_per_24h	6	0.000000	1.000000
LGBM vs. XGBoost	FA_per_24h	6	0.000000	0.125000
RF vs. XGBoost	FA_per_24h	6	0.000000	1.000000
Boosted vs. Bagged	FA_per_24h	18	12	0.137593

**Table 14:** Clinical equivalence testing against the 0.5 FA/24 h threshold.

Model	N	Mean FA/24 h	SD	95% CI	*p*-Value	Clinically Acceptable
CatBoost	6	0.001	0.002	[0.000, 0.002]	0.0000	✓
ET	6	0.000	0.000	[0.000, 0.000]	0.0000	✓
LGBM	6	0.002	0.002	[0.000, 0.004]	0.0000	✓
RF	6	0.000	0.001	[0.000, 0.001]	0.0000	✓
XGBoost	6	0.000	0.000	[0.000, 0.000]	0.0000	✓

**Table 15:** External validation performance on the Siena Scalp EEG Database (12 patients, 37 seizure events). Values are mean ± SD across patients.

Model	AUC	Event Sens.	Specificity	Events
Random Forest	**0.86 ± 0.11**	0.89 ± 0.21	**0.90 ± 0.08**	31/37 (84%)
Extra Trees	0.85 ± 0.12	**0.95 ± 0.14**	0.83 ± 0.10	**35/37 (95%)**
CatBoost	0.83 ± 0.17	0.89 ± 0.30	0.89 ± 0.09	32/37 (86%)
XGBoost	0.83 ± 0.18	0.65 ± 0.45	0.97 ± 0.04	24/37 (65%)
LightGBM	0.85 ± 0.16	0.59 ± 0.44	0.98 ± 0.04	20/37 (54%)

**Table 16:** Cross-dataset comparison: CHB–MIT (within-dataset) versus Siena (external validation).

Characteristic	CHB–MIT	Siena
Population	Pediatric (3–22 years)	Adult focal epilepsy
Recording site	Boston Children’s Hospital	University of Siena
Patients (seizure-positive)	6	12
Recording hours	∼180	∼125
Seizure events	∼20	37
Evaluation type	Within-dataset LOSO	External validation
**Best Model Performance**		
Event Sensitivity	95.0% (XGBoost)	94.9% (Extra Trees)
Segment-level AUC	0.997–0.998	0.85–0.86
Specificity	100%	83–90%

**Table 17:** Top SHAP features on Siena dataset (frequency across all models and patients).

Feature	Count	Channel Region
T3__skew	53	Left temporal
T4__abs_theta	34	Right temporal
O1__skew	34	Left occipital
T4__skew	28	Right temporal
Pz__skew	25	Parietal midline
T6__skew	24	Right posterior temporal
T4__hjorth_activity	20	Right temporal
T3__ptp	19	Left temporal

Temporal channels (T3, T4, T5, T6): 35% of all feature occurrences.

**Table 18:** Computational profiling of ensemble classifiers. Inference time measured per 5 s EEG segment (856 features) on Intel Core i9-12900K CPU without GPU acceleration. Real-time factor indicates processing speed relative to EEG acquisition rate (1× = processing one 5 s segment in 5 s).

Model	Memory (MB)	Inference (ms)	Throughput (seg/s)	Real-Time Factor
XGBoost	315.5	0.011	89,321	446,604×
LightGBM	25.8	0.014	73,661	368,307×
CatBoost	47.5	0.024	41,103	205,517×
Random Forest	63.5	0.154	6498	32,487×
Extra Trees	<1	0.155	6451	32,255×

## Data Availability

The original EEG datasets are openly available in PhysioNet: CHB-MIT Scalp EEG Database at (https://physionet.org/content/chbmit/1.0.0/, accessed on 18 January 2026) and Siena Scalp EEG Database at (https://physionet.org/content/siena-scalp-eeg/1.0.0/, accessed on 18 January 2026).

## Supplementary Materials

The following supporting information can be downloaded at https://www.mdpi.com/article/10.3390/s26030863/s1. Figure S1: Phase-specific EEG characteristics from patient chb01, showing raw traces, RMS amplitude topomaps, and theta-band power distributions across interictal, preictal, ictal, and postictal phases. Figure S2: Cross-model consensus heatmap of SHAP feature recurrence across all five ensemble models. Figure S3: Feature overlap analysis comparing common versus exclusive SHAP and LIME features. Figure S4: Rank-dispersion analysis of feature importance across folds and models. Table S1: Descriptive statistics per model across six patients under LOSO crossvalidation. Table S2: Inferential statistical tests on model performance and interpretability metrics. Table S3: Interpretability metric tests assessing SHAP-LIME Jaccard overlap and correlation with event sensitivity.
