# Supplementary material for: Explainable AI-Enhanced Ensemble Protocol Using Gradient-Boosted Models for Zero-False-Alarm Seizure Detection from EEG
Source: Sensors (Basel). 2026 Jan 28;26(3):863. doi: 10.3390/s26030863 (PMC12899994; doi:10.3390/s26030863)
Supplement: Supplementary file 1 [file sensors-26-00863-s001.zip › sensors-4018314-supplementary.pdf]

## Supplementary Materials

Supplementary Materials provide additional model validation, interpretability analyses, and phase-specific EEG characterizations. Figure S1 demonstrates electrophysiological progression across seizure phases. Figures S2–S4 show cross-model consensus on key biomarkers. Tables S1–S3 provide statistical validation of model performance and interpretability metrics.

- **Figure S1:** Phase-specific EEG characteristics from patient chb01, showing raw traces, RMS amplitude topomaps, and theta-band power distributions across interictal, preictal, ictal, and postictal phases.
- **Figure S2:** Cross-model consensus heatmap of SHAP feature recurrence across all five ensemble models.
- **Figure S3:** Feature overlap analysis comparing common versus exclusive SHAP and LIME features.
- **Figure S4:** Rank-dispersion analysis of feature importance across folds and models.
- **Table S1:** Descriptive statistics per model across six patients under LOSO cross-validation.
- **Table S2:** Inferential statistical tests on model performance and interpretability metrics.
- **Table S3:** Interpretability metric tests assessing SHAP-LIME Jaccard overlap and correlation with event sensitivity.

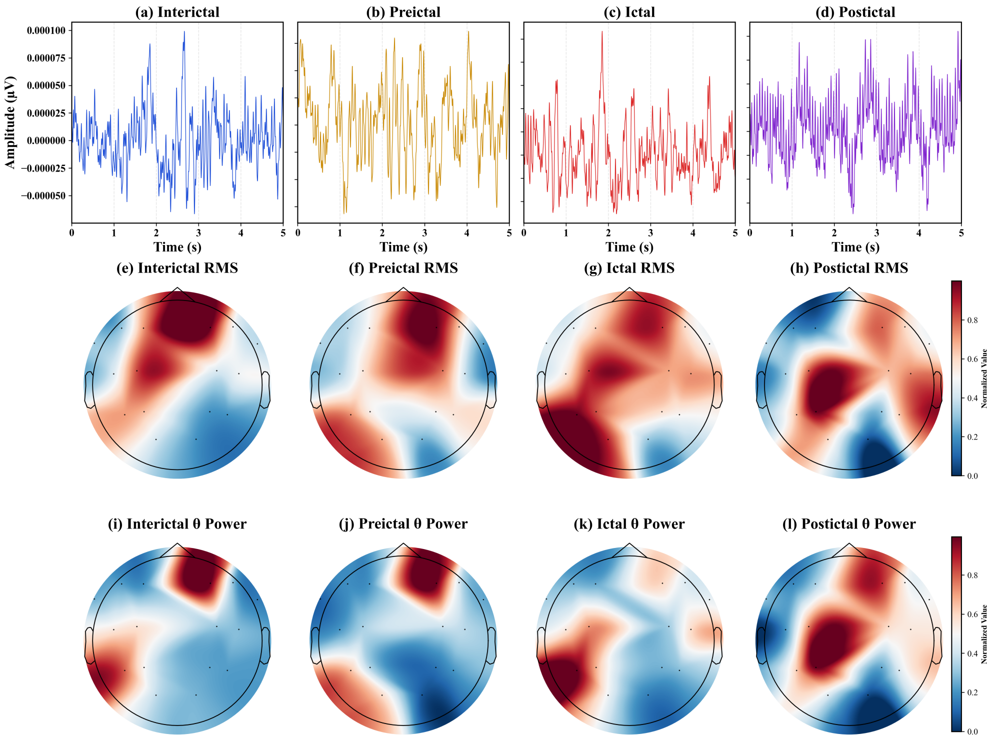


**Figure S1.** Phase-specific EEG characteristics from patient chb01. Top row (a–d): Five-second EEG segments from channel FT9-FT10 across four phases. (a) Interictal: baseline activity. (b) Preictal: 30 minutes before seizure onset. (c) Ictal: during seizure with pronounced theta-band rhythmicity. (d) Postictal: 30 minutes after seizure termination. Middle row (e–h): RMS amplitude topomaps showing progressive amplitude increase through ictal peak, followed by postictal decay. Bottom row (i–l): Theta-band (4–8 Hz) absolute power topomaps showing bilateral temporal activation during seizures, consistent with SHAP feature rankings (Table 5). All maps use standard 10–20 montage with values normalized to [0,1] within each phase.

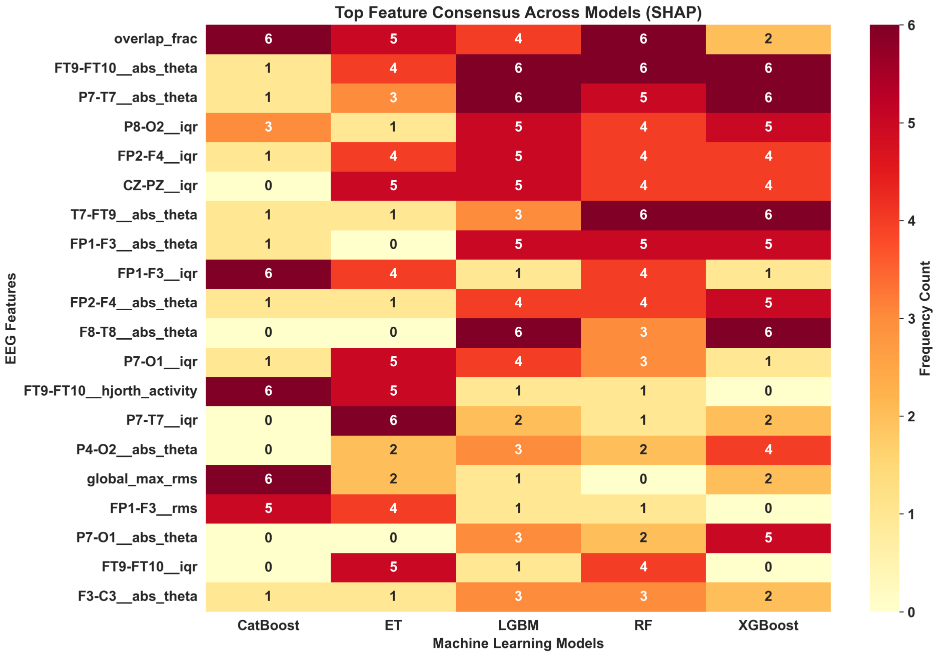


**Figure S2.** Cross-model consensus heatmap of SHAP feature recurrence across CatBoost, XGBoost, LightGBM, Random Forest, and Extra Trees. Higher counts indicate features consistently appearing in top-ranked sets across algorithms. Temporal theta-band power, frontal variability (FP1-F3__iqr), and RMS energy show the strongest cross-model agreement.

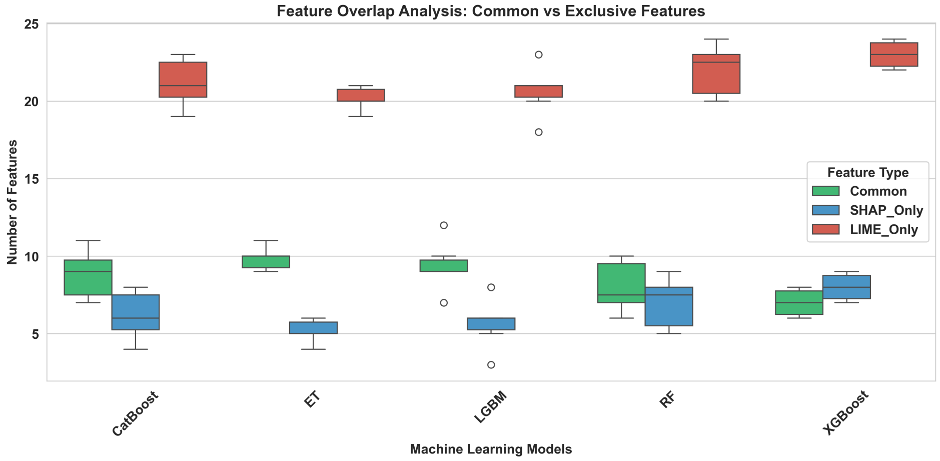


**Figure S3.** Feature overlap comparing SHAP and LIME features across models. Green bars denote features identified by both methods, blue those unique to SHAP, and red those unique to LIME. Approximately one-third of features overlap across folds, indicating a shared physiological core between global and local explanations.

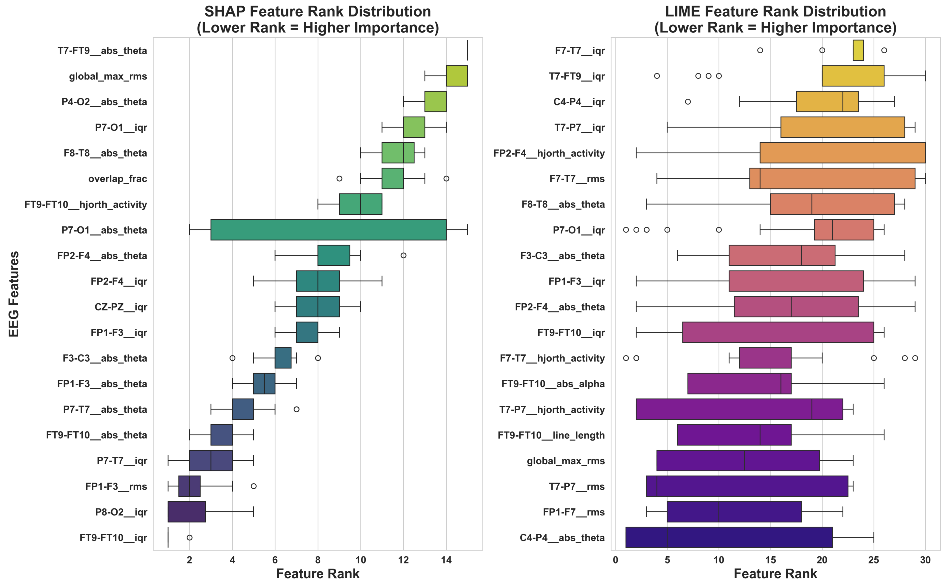


**Figure S4.** Rank-dispersion analysis of feature importance across folds and models. Narrower interquartile ranges for SHAP (left) indicate higher cross-fold stability relative to LIME (right).

**Table S1.** Descriptive statistics per model across six patients (LOSO). Values are Mean ± SD, Median [IQR], and 95% bootstrap CI.

| | Accuracy | | | Sensitivity | | | Specificity | | |
|---|---|---|---|---|---|---|---|---|---|
| Model | Mean±SD | Median[IQR] | 95% CI | Mean±SD | Median[IQR] | 95% CI | Mean±SD | Median[IQR] | 95% CI |
| CatBoost | 1.000 ± 0.000 | 1.000 [1.000, 1.000] | [1.000, 1.000] | 0.921 ± 0.126 | 0.986 [0.891, 1.000] | [0.816, 0.995] | 1.000 ± 0.000 | 1.000 [1.000, 1.000] | [1.000, 1.000] |
| ET | 1.000 ± 0.000 | 1.000 [1.000, 1.000] | [0.999, 1.000] | 0.893 ± 0.085 | 0.905 [0.862, 0.961] | [0.824, 0.947] | 1.000 ± 0.000 | 1.000 [1.000, 1.000] | [1.000, 1.000] |
| LGBM | 1.000 ± 0.000 | 1.000 [1.000, 1.000] | [0.999, 1.000] | 0.876 ± 0.127 | 0.891 [0.817, 0.978] | [0.773, 0.963] | 1.000 ± 0.000 | 1.000 [1.000, 1.000] | [1.000, 1.000] |
| RF | 0.999 ± 0.001 | 0.999 [0.999, 1.000] | [0.998, 1.000] | 0.591 ± 0.368 | 0.651 [0.421, 0.876] | [0.294, 0.839] | 1.000 ± 0.000 | 1.000 [1.000, 1.000] | [1.000, 1.000] |
| XGBoost | 1.000 ± 0.001 | 1.000 [1.000, 1.000] | [0.999, 1.000] | 0.922 ± 0.088 | 0.944 [0.856, 0.999] | [0.855, 0.981] | 1.000 ± 0.000 | 1.000 [1.000, 1.000] | [1.000, 1.000] |
| | Precision | | | F1 | | | AUC | | |
| Model | Mean±SD | Median[IQR] | 95% CI | Mean±SD | Median[IQR] | 95% CI | Mean±SD | Median[IQR] | 95% CI |
| CatBoost | 0.996 ± 0.007 | 1.000 [0.996, 1.000] | [0.990, 1.000] | 0.953 ± 0.073 | 0.988 [0.942, 0.996] | [0.893, 0.995] | 1.000 ± 0.000 | 1.000 [1.000, 1.000] | [1.000, 1.000] |
| ET | 1.000 ± 0.000 | 1.000 [1.000, 1.000] | [1.000, 1.000] | 0.941 ± 0.049 | 0.950 [0.926, 0.980] | [0.901, 0.972] | 1.000 ± 0.000 | 1.000 [1.000, 1.000] | [1.000, 1.000] |
| LGBM | 0.988 ± 0.013 | 0.991 [0.980, 0.999] | [0.978, 0.997] | 0.924 ± 0.074 | 0.941 [0.899, 0.977] | [0.864, 0.973] | 1.000 ± 0.000 | 1.000 [1.000, 1.000] | [1.000, 1.000] |
| RF | 0.831 ± 0.407 | 1.000 [0.990, 1.000] | [0.498, 1.000] | 0.672 ± 0.369 | 0.773 [0.592, 0.929] | [0.364, 0.898] | 1.000 ± 0.001 | 1.000 [1.000, 1.000] | [0.999, 1.000] |
| XGBoost | 1.000 ± 0.000 | 1.000 [1.000, 1.000] | [1.000, 1.000] | 0.958 ± 0.048 | 0.970 [0.922, 0.999] | [0.921, 0.990] | 1.000 ± 0.001 | 1.000 [1.000, 1.000] | [0.999, 1.000] |
| | FA_per_24h | | | | | | | | |
| Model | Mean±SD | Median[IQR] | 95% CI | | | | | | |
| CatBoost | 0.001 ± 0.002 | 0.000 [0.000, 0.001] | [0.000, 0.002] | | | | | | |
| ET | 0.000 ± 0.000 | 0.000 [0.000, 0.000] | [0.000, 0.000] | | | | | | |
| LGBM | 0.002 ± 0.002 | 0.001 [0.000, 0.002] | [0.000, 0.004] | | | | | | |
| RF | 0.000 ± 0.001 | 0.000 [0.000, 0.000] | [0.000, 0.001] | | | | | | |
| XGBoost | 0.000 ± 0.000 | 0.000 [0.000, 0.000] | [0.000, 0.000] | | | | | | |

**Table S2.** Inferential statistical tests on model performance and interpretability metrics.

| Test Type | Comparison | Metric | N | Statistic | p-value |
|---|---|---|---|---|---|
| Wilcoxon | CatBoost vs XGBoost | Sensitivity | 6 | 5.0000 | 1.0000 |
| Wilcoxon | ET vs XGBoost | Sensitivity | 6 | 5.0000 | 0.3125 |
| Wilcoxon | LGBM vs XGBoost | Sensitivity | 6 | 2.0000 | 0.1875 |
| Wilcoxon | RF vs XGBoost | Sensitivity | 6 | 0.0000 | 0.0312 |
| Wilcoxon | CatBoost vs XGBoost | AUC | 6 | 3.0000 | 1.0000 |
| Wilcoxon | ET vs XGBoost | AUC | 6 | 4.0000 | 0.8750 |
| Wilcoxon | LGBM vs XGBoost | AUC | 6 | 5.0000 | 0.6250 |
| Wilcoxon | RF vs XGBoost | AUC | 6 | 5.0000 | 0.6250 |
| Wilcoxon | CatBoost vs XGBoost | FA/24h | 6 | 0.0000 | 0.5000 |
| Wilcoxon | ET vs XGBoost | FA/24h | 6 | 0.0000 | 1.0000 |
| Wilcoxon | LGBM vs XGBoost | FA/24h | 6 | 0.0000 | 0.1250 |
| Wilcoxon | RF vs XGBoost | FA/24h | 6 | 0.0000 | 1.0000 |
| Mann-Whitney | Boosted vs Bagged | FA/24h | 30 | 134.5000 | 0.1376 |
| t-test | Jaccard vs 0.5 | Jaccard | 30 | -25.6547 | 0.0000 |
| Spearman | Jaccard vs Sensitivity | Correlation | 30 | $\rho = -0.1212$ | 0.5233 |

**Table S3.** Interpretability metric tests: SHAP-LIME Jaccard overlap and correlation with event sensitivity.

| Test | Comparison | N | Statistic | p-value |
|---|---|---|---|---|
| One-sample t-test | Jaccard vs 0.5 | 30 | -25.654726 | 0.000000 |
| Spearman correlation | Jaccard vs Event Sensitivity | 30 | $\rho$ = -0.121249 | 0.523312 |
